# Different origin-derived exosomes and their clinical advantages in cancer therapy

**DOI:** 10.3389/fimmu.2024.1401852

**Published:** 2024-06-27

**Authors:** Xiaoyan Jin, Jing Zhang, Yufu Zhang, Jing He, Mingming Wang, Yu Hei, Shutong Guo, Xiangrong Xu, Yusi Liu

**Affiliations:** ^1^ Department of Cell Biology and Genetics, Medical College of Yan’an University, Yan’an, Shaanxi, China; ^2^ The Second Affiliated Hospital of Xi‘an Medical University, Xi’an, Shaanxi, China; ^3^ Department of Hepatobiliary Surgery, The Affiliated Hospital of Yan’an University, Yan’an, Shaanxi, China; ^4^ Laboratory of Obstetrics and Gynecology, The Affiliated Hospital of Yan’an University, Yan’an, Shaanxi, China

**Keywords:** exosomes, cancer diagnosis, cancer treatment, biomarkers, targeted delivery

## Abstract

Exosomes, as a class of small extracellular vesicles closely related to the biological behavior of various types of tumors, are currently attracting research attention in cancer diagnosis and treatment. Regarding cancer diagnosis, the stability of their membrane structure and their wide distribution in body fluids render exosomes promising biomarkers. It is expected that exosome-based liquid biopsy will become an important tool for tumor diagnosis in the future. For cancer treatment, exosomes, as the “golden communicators” between cells, can be designed to deliver different drugs, aiming to achieve low-toxicity and low-immunogenicity targeted delivery. Signaling pathways related to exosome contents can also be used for safer and more effective immunotherapy against tumors. Exosomes are derived from a wide range of sources, and exhibit different biological characteristics as well as clinical application advantages in different cancer therapies. In this review, we analyzed the main sources of exosomes that have great potential and broad prospects in cancer diagnosis and therapy. Moreover, we compared their therapeutic advantages, providing new ideas for the clinical application of exosomes.

## 1 Introduction

In recent years, research has led to the continuous development of various technologies and the emergence of new drugs for the treatment of cancer worldwide (1–3). Nevertheless, therapeutic options fail to meet the clinical needs of patients, particularly those with recurrent or refractory cancer (4, 5). There are conflicting views regarding the appropriateness of various treatment options. For example, surgical resection is the most common treatment option for head and neck cancer (6–8). However, surgery is often unable to eradicate the tumor, leading to poor treatment effect (6). For glioma, the most common tumor type in the head and neck, surgical resection combined with temozolomide adjuvant chemotherapy is often used (9, 10); nonetheless, patients with glioma are prone to develop chemotherapy resistance (11, 12). Chemotherapy resistance is found in patients with various types of cancer (e.g., glioma, pancreatic, breast). Chemotherapy has limited effectiveness in the treatment of pancreatic and breast cancers (13, 14); thus, these cancer types are associated with high mortality rates (15). For the treatment of osteosarcoma, chemotherapy with doxorubicin has been linked to minimal success because of cardiac toxicity and limited drug targeting (16, 17), thereby complicating treatment (18, 19).

Traditional approaches to the treatment of tumors are characterized by several limitations. Therefore, new treatment methods have been gradually developed (e.g., immune checkpoint inhibitor therapy) (20, 21). Immune checkpoint inhibitors enhance the anti-cancer effect of treatment, thereby blocking the progression of tumors (22), especially in melanoma, lung cancer, and kidney cancer (23–25). However, the treatment is not effective against all tumor types (e.g., ovarian cancer, prostate cancer, pancreatic cancer, and glioblastoma) because certain tumors are “cold” due to the inability of immune cells to identify cancer cells (26, 27). This limits the effectiveness of immune checkpoint inhibitors, thus resulting in poor immune therapy outcomes (28, 29).

Immunotherapy-based cell therapies, such as mesenchymal stem cell (MSC) therapy, immune cell therapy (dendritic cells [DCs], natural killer [NK] cells, T cells, B cells, etc.) (30–32), and blood cell therapy (33), are also attracting considerable research attention for the treatment of cancer. Various macromolecules can be synthesized and secreted to exert paracrine effects and affect the local microenvironment, thus enhancing the effect of traditional surgical treatment (34, 35). This therapeutic approach can also locate the damage site, repair the damaged tissue, and achieve precise molecular targeting. However, cell therapy has been linked to risk of tumorigenicity, transmission, and unexpected differentiation (36). Moreover, due to the controlled regulation of the immune system, immunotherapy is often associated with severe adverse effects (e.g., autoimmune diseases, inflammation) (37). If immunotherapy is to move from preclinical research to clinical research, it is urgent to understand how to improve the response efficiency of different types of immunotherapy and avoid the risk of tumorigenicity, unexpected differentiation and inflammation (38). Due to the shortcomings of the above therapies, cell-free alternative therapies (e.g., gene therapy, exosome-based therapy) have attracted increasing attention (39–41). Cell-free therapies are relatively safe as compared with cell therapies and overcome the limitations of drug delivery to achieve effective penetration of target organs (42). Cell-free therapy can reduce the toxic side effects of radiotherapy and chemotherapy, as well as improve the patient’s own immunity and quality of life, This therapy is beneficial for almost all tumor types (43, 44). In recent years, with the gradual deepening of exosome research, the absolute advantages of exosomes as cell-free therapy have emerged (45). As a kind of natural extracellular vesicles, exosomes contain bioactive molecules for intracellular communication and intercellular material transport (46), which can be used as carriers to deliver small molecules, nucleic acids and other therapeutic drugs to the affected site (44), improve the local drug concentration and reduce side effects (47, 48). The low toxicity and low immunogenicity of exosome-mediated drug delivery provide hope for cell-free therapy of various diseases (47, 49). In addition, tumor-derived exosomes (TEX) play an important role in non-invasive liquid biopsy (50, 51). The discovery of TEX enables us to have a more comprehensive and specific understanding of exosomes, and also provides new ideas for clinical diagnosis and treatment (52).

**Figure 1:**
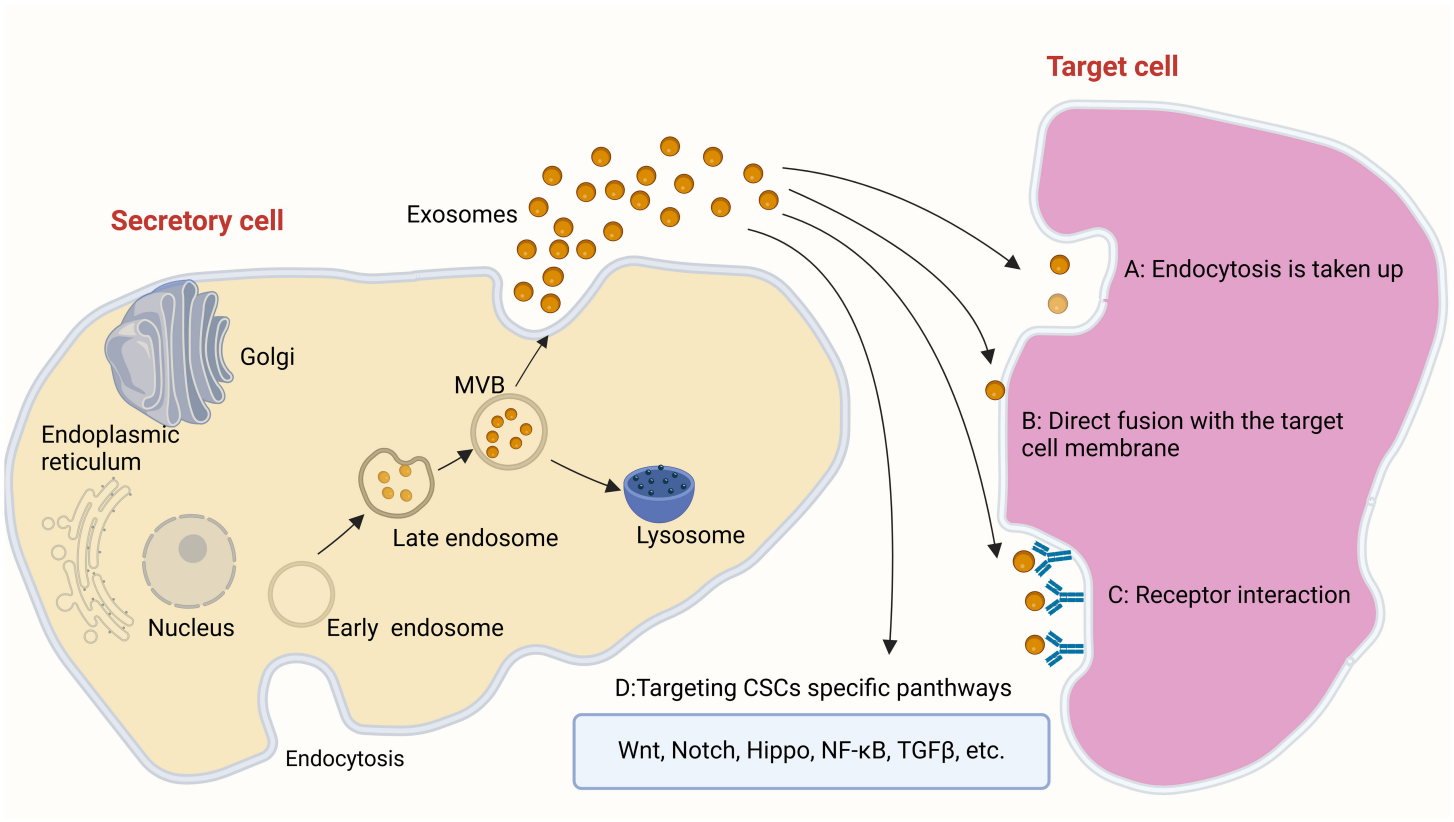
The way in which exosomes work: **(A)** Uptake by target cells via endocytosis **(B)** Direct fusion with the cell membrane of target cells **(C)** Receptor interaction D.Targeting CSCs specific panthways: Wnt, Notch, Hippo, NF-κB, TGFβ, etc.

Exosome-based therapy is a common method of cell-free therapy, that has shown great potential in inhibiting tumor progression or enhancing anti-tumor immunity (53). Studies have revealed that exosomes can easily cross biological barriers (e.g., blood–brain barrier [BBB] (54), skin mucosal barrier, placental barrier) and can be modified to improve their efficiency (55, 56). Due to the lipid bilayer structure, the unique surface, and their ability to transfer proteins, exosomes have been utilized as outside nanoparticle carriers of several drugs, nucleic acids, and protein receptors for various cancer cells (57, 58). Exosomes are a type of extracellular vesicles (EVs), along with microvesicles and apoptotic bodies (59). Microvesicles are 100–1,000 nm in size and are formed by cell membrane detachment following direct budding. Apoptotic bodies are protrusions (particle size: 1,000–5,000 nm) formed by the bubbled membrane of apoptotic cells during programmed cell death, which subsequently disintegrate (60). Exosomes (diameter: 50–150 nm) are classified as relatively small EVs. Exosomes are luminal vesicles (ILVs) that bud inward from the inner membrane during the maturation of multivesolar bodies (MVBs), namely early endosomes. After processing and modification, exosomes are formed into late endosomes (61). Exosomes secreted into the extracellular space act by binding to the corresponding recipient cells in four ways, including: A. Uptake by target cells via endocytosis, B. Direct fusion with the cell membrane of target cells, C. Receptor interaction, D.Targeting CSCs specific panthways: Wnt, Notch, Hippo, NF-κB, TGFβ, etc (62–65) (**Figure 1**). Exosomes contain various types of molecules (e.g., proteins, lipids, mRNA, DNA) (66). The molecular composition of exosomes is relatively stable and tissue-specific, and exosomes play important roles in cell communication (67). Exosomes exhibit tissue-targeting ability, good biocompatibility, low toxicity, low immunogenicity (47, 68, 69), and long-term stability and activity (47, 70). These advantages render exosomes ideal carriers for the delivery of anti-cancer drugs. Studies have shown that exosomes-coated drug (e.g., adriamycin, paclitaxel, sorafenib) can reduce the side effects of drugs, as well as improve treatment efficiency and drug utilization (71, 72).

**Figure 2:**
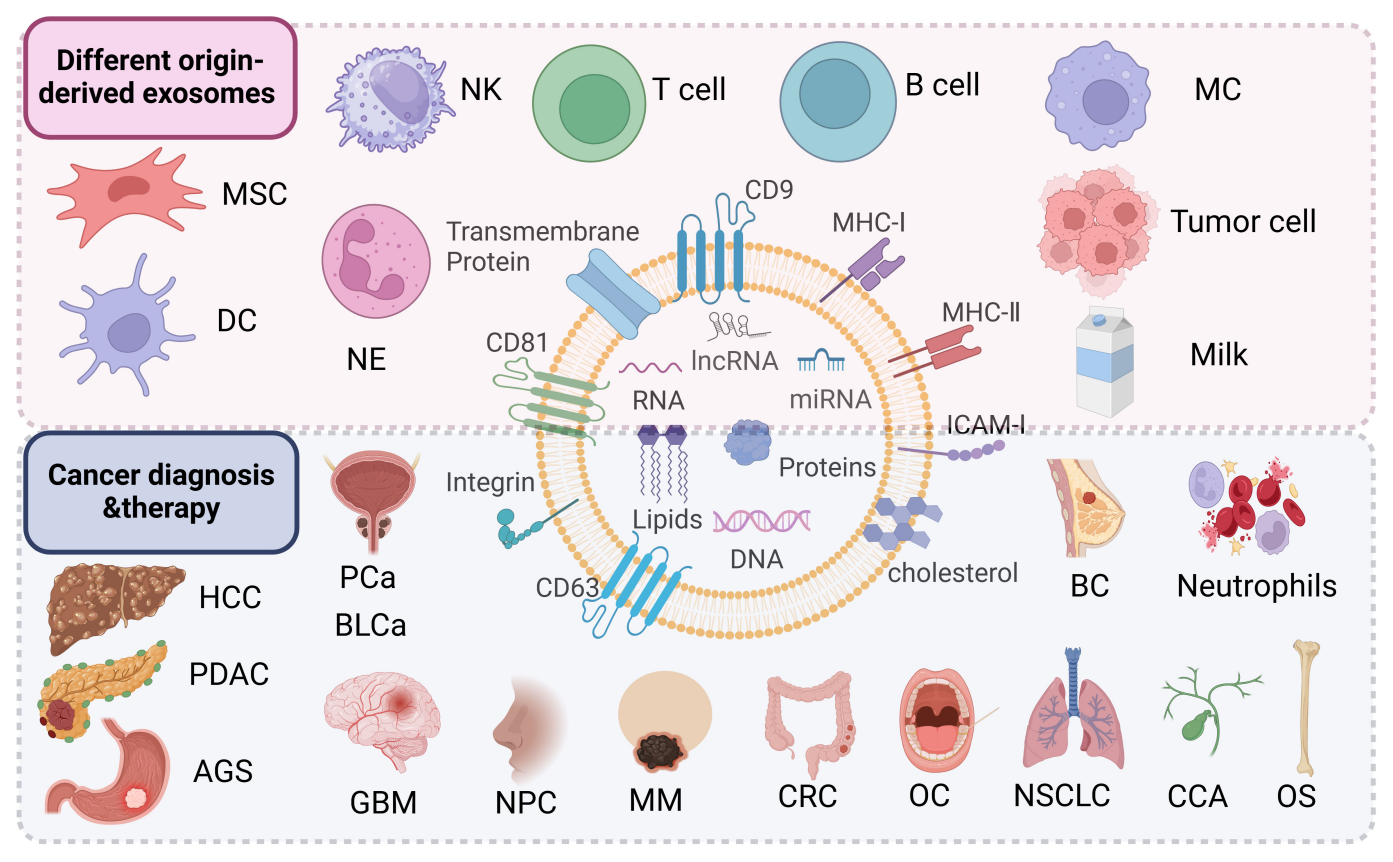
Exosomes derived from mesenchymal stem cells, immune cells (dendritic cells, macrophages, T cells, B cells, natural killer cells, neutrophils), tumor cells including hepatocellular carcinoma (HCC), pancreatic ductal adenocarcinoma (PDAC), gastric cancer (AGS), prostate cancer (PCa), bladder cancer (BLCa), glioblastoma (GBM), nasopharyngeal carcinoma (NPC), melanoma (MM), colorectal cancer (CRC), oral cancer (OC), non-small cell lung cancer (NSCLC), cholangiocarcinoma (CCA), osteosarcoma (OS), breast cancer (BC) and other sources are useful for the diagnosis and treatment of the company working.

Exosomes are derived from a wide variety of cell sources (73) (**Figure 2**). They can be obtained from the culture supernatant of MSCs, immune cells, cancer cells, epithelial cells, endothelial progenitor cells, platelets, and fibroblasts (74, 75). Furthermore, they can also be found in various body fluids (e.g., blood, urine, breast milk, saliva) (76). Exosomes have been used as cell-free therapy in multiple manners, and are promising biomarkers for cancer diagnosis and prognosis (77, 78). In addition, exosomes derived from different sources show multi-dimensional features and functions, providing new ideas for the diagnosis and treatment of various cancer types (79, 80).

## 2 MSCs-derived exosomes (MSCs-Exo) in cancer therapy

MSCs are adult stem cells with potential for self-renewal and multi-directional differentiation (81). They can be isolated from bone marrow, fat, umbilical cord, dental pulp, and numerous other tissues (82, 83). Under appropriate conditions, MSCs can differentiate into various cell types (e.g., osteoblasts, adipocytes, chondrocytes) (84, 85). They possess significant anti-inflammatory properties and play important roles in immune regulation (86), hematopoiesis, and tissue repair (87–89). Studies have shown that MSCs have tumor tropism ability, enabling them to offer unique advantages in tumor therapy and regulate tumor fate (90).

Recently, MSCs-Exo have been shown to act as a novel drug delivery system to package various target molecules and play a therapeutic role in various diseases (91). They also play an active role in the process of vascular development and repair in multiple tissues (92, 93). MSCs-Exo are characterized by low immunogenicity (94, 95), high biocompatibility (95, 96), and high stability as a carrier. These features offer a new option for the delivery of drugs targeting tumor cells (97). As an ideal drug delivery system, MSCs-Exo can selectively deliver therapeutic drugs to the target, avoid recognition and degradation by immune cells, and control the release of combined therapeutic drugs at the target. Modified MSCs-Exo are obtained by incorporating different therapeutic agents (e.g., proteins, RNA, chemotherapy drugs) into MSCs-Exo through several loading methods (98). At present, these drugs have been loaded into exosomes by ultrasonic treatment, electroporation, transfection, incubation, extrusion, saponin-assisted loading, transgenic, freeze-thaw cycle, heat shock, pH gradient method, and hypoosmotic chromatography (99). It has been shown that modified MSCs-Exo improve the therapeutic efficacy of cancer. Different MSCs-Exo can also offer their outstanding advantages as drug delivery systems. Despite the great potential of MSCs-Exo, their application as a drug delivery system has been hampered by several challenges (100). Hence, the clinical application of modified Exo warrants further investigation.

### 2.1 Exosomes from human umbilical cord-derived MSCs (hucMSCs)

**Figure 3:**
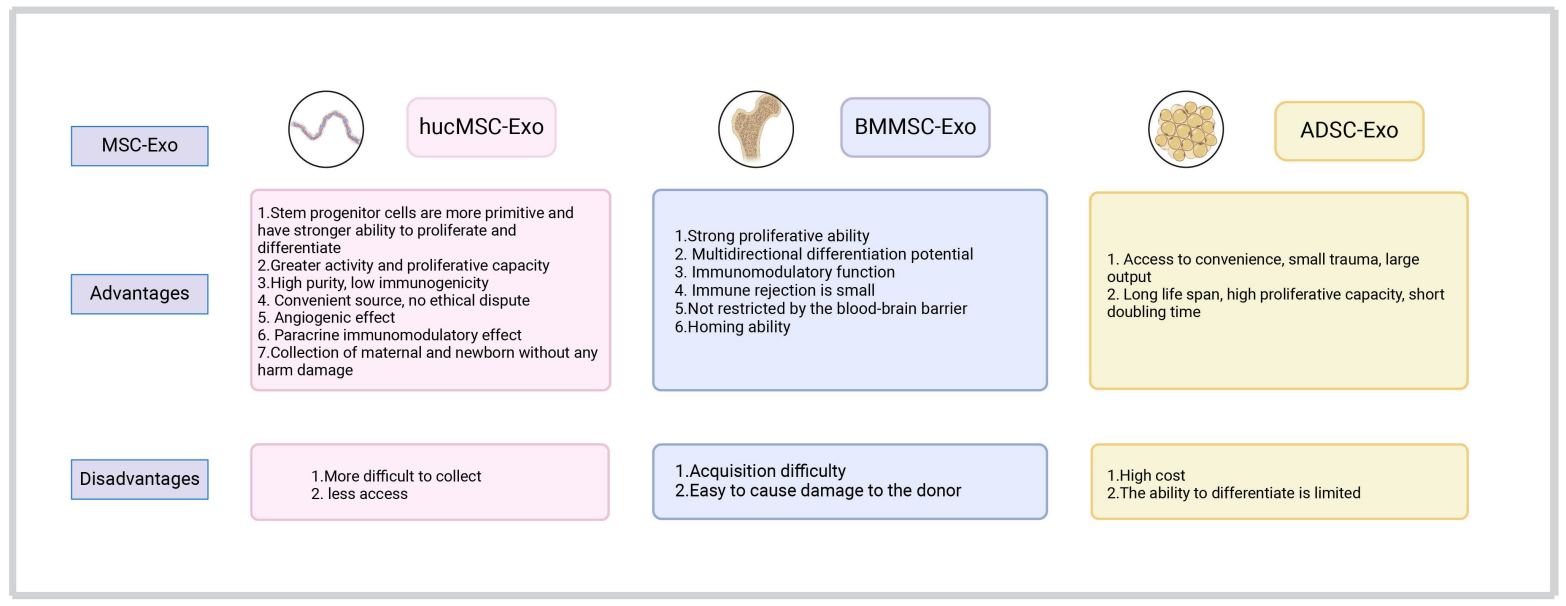
A comparison of the advantages and disadvantages of exosomes derived from umbilical cord, bone marrow, and adipose-derived mesenchymal stem cells in clinical application.

The human umbilical cord is a promising source of MSCs (101). Different from bone marrow stem cells and adipose-derived stem cells (ADSCs), hucMSCs have been associated with painless collection, easy acquisition (102), more primitive cells, higher proliferative ability, less immune rejection (103), and ability for faster self-renewal (104) (**Figure 3**). The hucMSCs differentiate into various cells in three germ layers (e.g., bone, cartilage, fat, skeletal muscle, myocardial cells, endothelial cells), and synthesize and secrete a group of trophic factors and cytokines (103). They also support the expansion and function of other cells (e.g., hematopoietic stem cells, embryonic stem cells, NK cells, islet-like cell clusters, neurons, and glial cells), and can migrate to and return to the pathological area (105). Evidence has shown that hucMSCs-derived exosomes (hucMSCs-Exo) have similar functions to hucMSCs, with low immunogenicity and non-tumorigenicity (102). As a new cell-free alternative therapy, hucMSCs-Exo have been widely used in regenerative medicine and cancer treatment (106, 107). Below, we introduce the application of hucMSCs-Exo in the treatment and diagnosis of cancer in various systems of the body.

#### 2.1.1 hucMSCs-Exo for head and neck tumors

**Figure 4:**
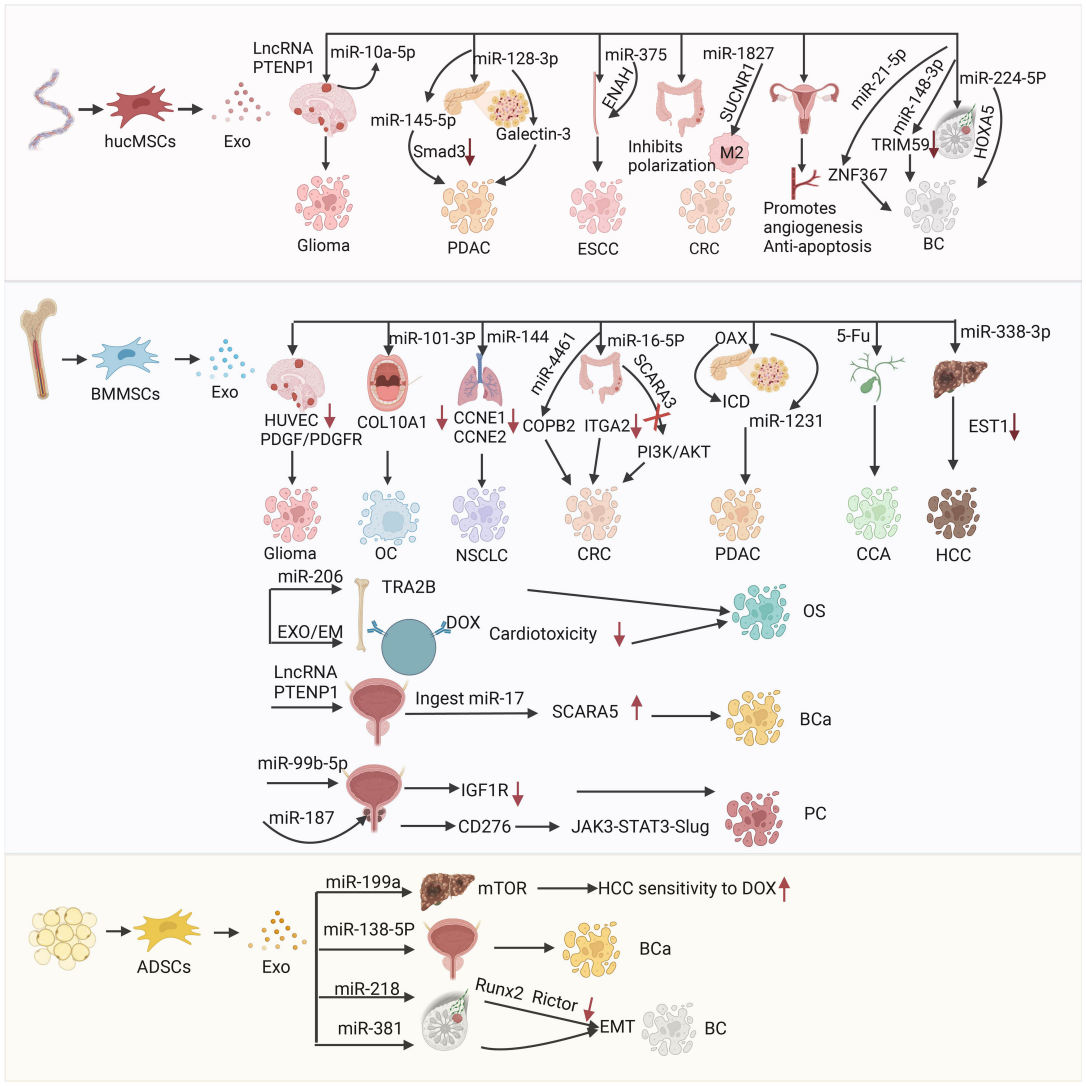
The mechanism of action and therapeutic targets of hucMSCs-Exo in the treatment of glioblastoma(GBM), pancreatic ductal adenocarcinoma (PDAC), esophageal squamous cell carcinoma (ESCC), colorectal cancer (CRC), and breast cancer (BC); the mechanism of action and targets for therapy using BMMSCs-Exo in the treatment of glioblastoma (GBM), oral cancer (OC), non-small cell lung cancer (NSCLC), colorectal cancer (CRC), pancreatic ductal adenocarcinoma (PDAC), cholangiocarcinoma (CCA), hepatocellular carcinoma (HCC), osteosarcoma (OS) and bladder cancer (BCa); the mechanism of action for ADSCs-Exo in treating hepatocellular carcinoma (HCC), bladder cancer (BCa) and breast cancer (BC).

Brain tumors are among the deadliest types of cancer (108, 109). Great progress has been achieved in early diagnosis and treatment (e.g., surgical resection, adjuvant radiotherapy, and chemotherapy) (110). Nevertheless, the prognosis of patients with glioma remains poor and the mortality rate is high due to the lack of radical treatment (111–113). Moreover, the BBB prevents the complete delivery of drugs to the brain tissue; thus, the treatment of brain tumors is challenging (114). Studies have shown that exosomes contain various long-noncoding RNAs (lncRNAs) and proteins (115), which are involved in intercellular communication and cell signal transduction (40). For example, lncRNA phosphatase and tensin homolog pseudogene 1 (PTENP1) is a competing endogenous RNA, which exerts its tumor suppressor function by regulating the expression of PTEN in many malignant tumors (116). MicroRNAs (miRNAs) are small noncoding RNA molecules that regulate gene expression (117). According to their gene targets, miRNAs have been associated with cancer development and oncogenic (or tumor suppressor) effects (118). The miRNAs play a role in almost all aspects of cancer biology (e.g., proliferation, apoptosis, invasion/metastasis, angiogenesis) (118). There are many types of miRNA (e.g., miR-155, miR-10b, miR-21, miR-10a, miR-10a-5p, miR-221) (119). Among them, miR-10a-5p can promote the progression of pancreatic cancer, bladder cancer, cholangiocarcinoma, and other tumors (120–122). Hao et al. investigated the mechanism of lncRNA PTENP1 in glioma. They found that lncRNA PTENP1 could be packaged into hucMSC-Exo and transferred to glioma cell line U87 cells, where it bound to miR-10a-5p in tumor tissues (123) (**Figure 4**). Thus, it inhibits the expression of tumor suppressor gene PTEN and prevents tumor progression. The results showed that hucMSCs-Exo have high anti-tumor ability by regulating the miR-10a-5p/PTEN signaling pathway (123). This evidence may provide a possible target for the early diagnosis and treatment of glioma in clinical practice (123).

#### 2.1.2 hucMSCs-Exo for gastrointestinal tumors

Pancreatic cancer is an insidious and highly metastatic malignant tumor type (124, 125), which progresses very rapidly (126); the 5-year survival rate of patients with pancreatic cancer is <10% (127, 128). Pancreatic ductal adenocarcinoma (PDAC) accounts for >90% of pancreatic cancer cases (129), and is one of the most aggressive types of tumors worldwide with a very poor prognosis (130). Chemotherapy is currently the first-line treatment for pancreatic cancer in clinical practice (131); however, its therapeutic effect is poor due to the existence of chemoresistance mechanisms (132).

Exosomes are important mediators of intercellular communication in the development of drug resistance, and can be used as delivery tools (122, 133). They have become a key carrier to deliver miRNA to cancer cells (70, 134), and their function is often achieved through pathways related to transforming growth factor-β (TGF-β) (135). TGF-β is a member of the TGF-β family of growth and differentiation factors, which consists of TGF-β, bone morphogenetic proteins (BMPs), TGF-βs, growth and differentiation factors (GDFs), activin/inhibin, mullerian inhibitory factor (MIF), and other structural-related protein family (136). TGF-β is highly associated with cell proliferation and differentiation (137), immune surveillance (138), inflammation, and cancer development (139). However, overexpression of TGF-β can cause the formation of cancer-associated fibroblasts, extracellular matrix, and epithelial–mesenchymal transition (EMT), which could lead to cancer (139). TGF-β pathway plays a dual role in cancer progression (140, 141); it is an inhibitor of tumor cell growth and an inducer of tumor metastasis (142), thereby playing an important role in the development and metastasis of tumors (143). TGF-β functions mainly by binding to the corresponding ligands, which are divided into three main classes, namely cell surface type I and type II serine/serine kinase receptors (TGFβRI and TGFβRII, respectively) and co-receptors endobilin and β-glycans (termed type III receptors or TGFβRIII) (144). It acts by binding to its corresponding receptors and activating the downstream effector molecule Smad, which is the core of the TGF-β pathway and the key intracellular effector of TGF-β (129, 145). TGF-β/Smad signaling plays an important role in fibrosis, and elevated TGF-β levels in serum or tumor tissues indicate poor prognosis of PDAC, Ding et al. demonstrated that hucMSCs-Exo could transfer exogenous tumor suppressor miR-145–5p (146). The hucMSCs-Exo could downregulate the expression of Smad3 in PDAC cells and inhibit the proliferation and invasion of PDAC cells. These findings also indicated that hucMSCs-Exo could be an excellent delivery vector for exogenous miR-145–5p to inhibit the progression of PDAC (146). Galectin-3 (LGALS3) is a member of the galectin family (147). Galectins are located on the membrane of various tumor cells and participate in the regulation of cell growth, inhibition of cell apoptosis, and mediation of cell adhesion, as well as the formation of new blood vessels, and tumor invasion and metastasis (148) (**Figure 4**). Therefore, LGALS3 shows promise as a therapeutic target for pancreatic cancer. Xie et al. reported that hucMSCs-Exo could carry miRNA-128–3p to inhibit the proliferation, invasion, and migration of PANC-1 cells *in vitro* by targeting LGALS3 through miRNA (149).

The hucMSCs-Exo also play a great role in the treatment of colorectal cancer (CRC) and esophageal cancer (107). CRC is the second most common type of cancer globally (150), and its etiology includes genetic and environmental factors (151, 152). Treatment includes endoscopic and surgical local excision, preoperative radiotherapy and systemic therapy, targeted therapy, and immunotherapy (153, 154). Succinic acid receptor 1 (SUCNR1) mutation is a gene mutation causing rectal and gastric cancer. This gene promotes lung cancer metastasis by promoting macrophage polarization. Therefore, targeting SUCNR1 may be a promising approach to CRC treatment. Cell-free alternative therapy has been gradually applied to CRC (155). Chen et al. found that hucMSCs-Exo enriched with miR-1827 plays an important role in inhibiting liver metastasis of CRC by targeting SUCNR1 to inhibit M2 macrophage polarization (**Figure 4**). These exosomes can inhibit the progression and metastasis of CRC (156).

Esophageal cancer remains one of the most prevalent and aggressive types of cancer (157). Clinically, there are two subtypes of esophageal cancer, namely esophageal adenocarcinoma and esophageal squamous cell carcinoma (ESCC) (158). Downregulation of miRNA-375 is a common phenomenon in ESCC, and associated with poor prognosis, low survival rate, and tumor metastasis (159). Using bioinformatics databases, He et al. predicted the target-enabled homolog of miRNA-375 (ENAH), commonly known as MENA, This is a member of the Ena/vasodilator stimulated phosphoprotein (Ena/VASP) group and consists of actin-related proteins that play diverse roles in different cells. The hucMSCs-Exo delivered miRNA-375, which combined with ENAH inhibited ESCC cell proliferation, invasion, and migration, and promoted cell apoptosis and tumor growth (160).

#### 2.1.3 hucMSCs-Exo for reproductive system tumors

Ovarian and breast tumors are the main types of reproductive system cancer that threaten the life and health of women (161). Ovarian cancer refers to a group of heterogeneous tumors that can originate from any histological part of the ovary (e.g., epithelial cells, stromal cells, and germ cells) (162). The treatment of ovarian cancer includes surgery and chemotherapy. Despite aggressive treatment, the survival rate of patients with advanced ovarian cancer remains poor (163). Thus, more effective methods of diagnosis and treatment are needed (164). Qu et al. found that hucMSCs-Exo could be used to carry miR-126–3p, forming miR-126–3p-hucMSCs-Exo. Notably, miR-126–3p was a positive regulator of angiogenic activity. In the treatment of premature ovarian cancer, miR-126–3p promotes ovarian angiogenesis and anti-apoptosis (165).

The hucMSCs-Exo have also been utilized in breast cancer therapy. Breast cancer is the most common type of cancer in women, the second most common type among newly diagnosed cancers worldwide, and the leading cause of cancer-related death (166). It is a heterogeneous disease involving genetic and environmental factors. The treatment methods include surgery, radiotherapy, and chemotherapy. Despite the continuous improvement of therapeutic methods, drug resistance remains a great obstacle. New targeted therapies provide novel ideas for the treatment and diagnosis of breast cancer. Exosomal miR-21–5p is significantly upregulated and promotes metastasis in several types of cancer; however, its role in breast cancer has not been thoroughly investigated. Du et al. found that miR-21–5p can be used in the treatment of breast cancer (167). Zinc finger protein 367 (ZNF367) belongs to the zinc finger protein family and is overexpressed in various types of cancer. ZNF367 inhibited tumor growth, proliferation, migration, and invasion of breast cancer, promoting tumor invasion and metastasis. The miR-21–5p carried by hucMSCs-Exo binds to the 3’-untranslated region (3’-UTR) of ZNF367 to inhibit the progression of breast cancer (167). The hucMSC-Exo carried miR-224–5P and miR-148b-3p, which also played an important role in inhibiting the progression of breast cancer. Wang et al. found that hucMSCs-Exo carrying miR-224–5p played a role in autophagy in breast cancer, and miR-224–5p could target and bind to stem cell-related gene (homeobox A5 [HOXA5]) to regulate autophagy (168). Moreover, it can affect the proliferation and apoptosis of breast cancer cells. Yuan et al. reported that hucMSCs-Exo carrying miR-148b-3p inhibited the progression of breast cancer by downregulating tripartite motif containing 59 (TRIM59). The latter is related to the regulation of the development of human diseases (e.g., cancer). Elevated TRIM59 has also been detected in numerous malignancies, including breast cancer (169). Downregulation of TRIM59 inhibits the progression of breast cancer (**Figure 4**).

### 2.2 Exosomes from bone marrow-derived MSCs (BMMSCs)

BMMSCs are the first MSCs identified (84). They have been described as the progeny of fibroblasts possessing colony-forming ability and differentiation potential (170, 171). In addition to BMMSCs-specific markers (CD73, CD90, CD105) and negative surface markers (CD11b, CD14, CD19, CD34, CD45, CD79a), and human leukocyte antigen-DR (HLA-DR), human-derived BMMSCs also express other markers (CD10, CD29, CD44, CD133) (171). BMMSCs are multipotent cells that can differentiate into osteoblasts, chondrocytes, and adipocytes (172). They are widely used in the treatment of various diseases due to their self-regeneration, differentiation, and immune regulation (downregulation of T cells, B cells, NK cells, and antigen-presenting cells through various mechanisms) (173). Compared with hucMSCs and ADMSCs, BMMSCs have high potency for clinical use in the treatment of various diseases (e.g., bone and cartilage, immune system, nervous system, cardiovascular, viral/infectious, cancer, wounds and injuries). The exosomes produced by BMMSCs can also be utilized for this purpose (174, 175). Compared with BMMSCs, BMMSCs-derived exosome (BMMSCs-Exo) have smaller volume, are associated with less immune rejection, and can easily transport therapeutic agents, thus playing a great role in overcoming resistance to cancer treatment (**Figure 3**). Consequently, BMMSCs-Exo show promise in the treatment of cancer.

#### 2.2.1 BMMSCs-Exo for head and neck tumors

Anti-angiogenesis strategies are often used in the treatment of glioma (176). These strategies mainly target the vascular endothelial growth factor (VEGF) signaling pathway (VEGF/VEGFR) (177), angiopoietin/Tie2 (Ang/Tie2) signaling pathway, and matrix metalloproteinases (MMPs) (178, 179). It is well established that the platelet-derived growth factor/platelet-derived growth factor receptor (PDGF/PDGFR) axis also plays a key role in glioma angiogenesis (180). Han et al. reported that BMMSCs*-*Exo could inhibit the growth of glioma cells *in vitro* and *in vivo*. After co-culture of BMMSCs-Exo and glioma cells, the number of endothelial progenitor cells and human umbilical vein endothelial cells was reduced, and the angiogenesis ability was weakened (181). The underlying mechanisms were reduced levels of PDGF-BB, interleukin-1 (IL-1), phosphorylated-protein kinase B (p-AKT) and cathepsin B (CTSB). These exosomes exert their anti-tumor effects by downregulating the PDGF/PDGFR axis (181).

Oral cancer is currently the sixth most common type of malignant tumors worldwide, threatening the health of individuals (182). The treatment of oral cancer includes traditional (surgery, radiotherapy, and chemotherapy) and new (photothermal therapy, exosomes) options. In exosome therapy, exosomes are often used to carry miRNA (183). Studies have shown that the disorder of miRNA is related to the malignant transformation of tumors (184). For example, miRNA-585 is lowly expressed in oral cancer and can be used as a tumor suppressor, Shah et al. found that the expression of miRNA21 was negatively correlated with the prognosis of oral cancer (185). Xie et al. used a nano-miRNA system to achieve targeted therapy of oral cancer. Exosomes delivered miR-101–3p *in vitro* and *in vivo* to inhibit the proliferation, invasion, and migration of oral cancer cell line TCA8113, as well as inhibit tumor growth by targeting and downregulating collagen type X alpha 1 chain (COL10A1) of the collagen family (186). Therefore, BMMSCs-Exo upregulating miR-101–3p may become a new direction for the development of oral cancer treatment (186). However, the underlying mechanisms require further investigation (**Figure 4**).

#### 2.2.2 BMMSCs-Exo for respiratory tumors

Cyclin E1 (CCNE1) is an oncogenic driver gene that promotes the progression of various cancer types (e.g., lung, ovarian, endometrial) (187). CCNE2 protein forms a complex with cyclin dependent kinase 1 (CDK1), promoting cell cycle switching from G1 to S phase (188). Liang et al. reported that CCNE1 and CCNE2 can be used as therapeutic targets for non-small cell lung cancer, and BMMSCs-Exo carrying miR-144 could target CCNE1 and CCNE2. Downregulation of CCNE1 and CCNE2 can inhibit the development of non-small cell lung cancer cells (189).

#### 2.2.3 BMMSCs-Exo for gastrointestinal tumors

The miR-4461 contained in BMMSCs-Exo targets envelope coatomer protein complex β2 (COPB2) and inhibits the migration and invasion of CRC cells. BMMSCs-Exo overexpressing miR-16–5p inhibit the proliferation, migration, and invasion of CRC cells (115). Moreover, they stimulate the apoptosis of CRC cells by downregulating integrin-α2 (ITGA2) (190). Scavenger receptor class A member 5 (SCARA5) is a newly discovered tumor suppressor which inhibits the phosphorylation of AKT and phosphatidylinositol 3-kinase (PI3K) in CRC cells and tumors. Notably, SCARA5 in BMMSCs-Exo inhibits CRC progression by inactivating PI3K/AKT (191, 192). This evidence highlights the potential clinical utility of SCARA5-containing BMMSCs-Exo in the treatment of CRC (192). BMMSCs-Exo may also be used in the treatment of colitis. Moreover, the effect of interferon-γ-induced (IFN-γ-induced) BMMSCs-Exo in this setting was obvious. IFN-γ directly targets and inhibits signal transducer and activator of transcription 3 (STAT3) by upregulating the expression of miR-125a and miR-125b, thereby inhibiting T helper 17 (Th17) differentiation and enhancing the ability of BMMSCs-Exo to improve the colitis phenotype in mice (193).

The role of BMMSCs-Exo in the treatment of pancreatic cancer cannot be ignored. BMMSCs-Exo significantly inhibited the invasion, migration, and proliferation of PDAC cells, as well as tumor stemness (194). Exosomes extracted from BMMSCs with high levels of miR-1231 inhibit the activity of PDAC, and exosomal miR-1231 may also be a potential indicator for the diagnosis of pancreatic cancer in the future (195). The induction of more intratumoral effector immune cells and the reversal of immunosuppression are the key to the treatment of PDAC (196). BMMSCs-Exo delivery system was constructed using oxaliplatin prodrug surface modification as an immunogenic cell death trigger. BMMSCs-Exo were used to improve PDAC-targeting ability and increase drug accumulation in PDAC cells (197).

In addition to the construction of a nano-miRNA system, exosomes are often used to deliver chemotherapy drugs (e.g., doxorubicin, paclitaxel, curcumin, temozolomide, 5-fluorouracil [5-FU]) (198). The delivery of chemotherapeutic drugs can reduce drug resistance and the toxicity of direct use of chemotherapeutic drugs, as well as achieve more targeted therapy and improve the utilization of drugs (199). Chen et al. reported that the anti-cholangiocarcinoma drug 5-FU was loaded into BMMSCs-Exo using sonication and incubation methods. The anti-tumor activity of 5-FU-BMMSCs-Exo was higher than that of free 5-FU. BMMSCs-Exo-delivered 5-FU can combat cholangiocarcinoma *in vitro*, achieving targeted delivery (200).

Li et al. investigated the role of exosomal miR-338–3p derived from BMMSCs in hepatocellular carcinoma (HCC). They found that exosomal miR-338–3p upregulation or EST1 silencing inhibited the proliferation, invasion, and migration of HCC cells, and induced apoptosis (201). BMMSCs-Exo-delivered miR-338–3p can delay the development of HCC by targeting and downregulating EST1, thus providing a new promising therapeutic target for HCC (202).

#### 2.2.4 BMMSCs-Exo for skeletal system tumors

Osteosarcoma is a type of bone tumors with a high incidence in children and adolescents (203, 204). It has been reported that transformer 2 beta homolog (TRA2B) is overexpressed during the progression of osteosarcoma, and BMMSCs-Exo can carry miR-206 and target TRA2B to inhibit the progression of this disease (205). Wei et al. also found that BMMSCs-Exo carried chemotherapy drug doxorubicin to treat osteosarcoma. Moreover, use of the nano-drug delivery system reduced the cardiotoxicity of treatment with doxorubicin and improved its targeting effect (206). BMMSCs exosome mimetic was prepared, and doxorubicin was embedded into it to form a complex for the treatment of osteosarcoma (206). The exosome mimetic-doxorubicin showed more potent tumor inhibitory activity and fewer side effects than free doxorubicin. This novel bio-nanomedicine system may provide a good strategy for the development of novel precision drugs for osteosarcoma.

#### 2.2.5 BMMSCs-Exo for urologic tumors

Bladder cancer is the most common malignant tumor type in the urinary tract. Surgical resection is often used for the treatment of bladder cancer (207). In recent years, with the continuous development of nanotechnology, new ideas for the treatment of bladder cancer have been reported. LncRNA PTENP1 is a competing endogenous RNA (208). It has been reported that lncRNA can be transferred to tumor cells through BMMSCs-Exo, Liu et al. found that BMMSCs-derived exosomal lncRNA PTENP1 inhibited the progression of bladder cancer by upregulating SCARA5 expression through miR-17 uptake (209). Exosomes derived from PTENP1-overexpressing BMMSCs abolished the promotion of miR-17 overexpression or SCARA5 knockdown on the malignant phenotype of bladder cancer cells. It has also been shown that they inhibit the growth of bladder cancer tumors in nude mice *in vivo*. This effect is achieved through the miR-17/SCARA5 axis (209). These data provide a potential new therapeutic target for the treatment of bladder cancer.

Prostate cancer is the most common type of cancer in men worldwide (210). Patients with advanced or metastatic prostate cancer expire due to the disease even after therapeutic interventions (e.g., radiotherapy, surgery, androgen deprivation therapy, chemotherapy) (211). Malla et al. reported that miR-99b-5p is enriched in serum exosomes of patients with prostate cancer undergoing radiotherapy (212). Moreover, Jiang et al. reported that miR-99b-5p mimics or inhibitors were transfected into BMMSCs-Exo, and prostate cancer cell line LNCaP cells were stimulated using BMMSCs-Exo with miR-99b-5p (213). It was found that BMMSCs-Exo significantly inhibited the malignant phenotype of prostate cancer cells, and transfection of BMMSCs with a miR-99b-5p mimic further enhanced the inhibitory effect on the progression of prostate cancer, Transfection of BMMSCs-Exo with a miR-99b-5p inhibitor promoted prostate cancer progression *in vitro (* 213). Further studies on the mechanism underlying the inhibitory effect on prostate cancer found that miR-99b-5p could bind to its downstream target insulin-like growth factor 1 receptor (IGF1R), downregulate it, and inhibit the progression of prostate cancer. BMMSCs could attenuate the progression of prostate cancer, and exosomal miR-99b-5p and IGF1R were involved in the regulatory process. This evidence contributes to our understanding of the pathogenic mechanism of prostate cancer. Li et al. also reported the function of BMMSCs-Exo carrying miR-187 in prostate cancer (214). Of note, miR-187 can be used as the main diagnostic marker of metastatic prostate cancer (215). Studies have shown that upregulation of miR-187 leads to decreased CD276 expression (B7 homologue 3 protein, namely B7-H3, a new member of B7 family immunoregulatory proteins and a promising target for cancer immunotherapy) and inhibits the Janus kinase 3/STAT3/SLUG (JAK3/STAT3/SLUG) signaling pathway, It has been demonstrated that BMMSCs-Exo carrying miR-187 can inhibit the progression of prostate cancer through targeting CD276 and the JAK3/STAT3/SLUG axis (**Figure 4**).

### 2.3 ADSCs-derived exosomes (ADSCs-Exo)

ADSCs exhibit positivity for tumor susceptibility 101 (TSG101), CD63, CD9, CD13, CD29, CD44, CD73, CD90, and CD105 (216); in contrast, they show negativity for calnexin (CANX), CD31, and CD45 (217). Compared with BMMSCs, ADSCs have a longer life span, higher proliferative ability, shorter doubling time, and later senescence *in vitro (* 218). Furthermore, the collection of ADSCs is more convenient and less invasive, while the yield is larger. Although ADSCs offer multiple advantages, their clinical application is limited due to the possible promotion of tumor development (**Figure 3**). Subsequent research revealed that the promotive effect of ADSCs on cancer is attributed to the adipose tissue around the tumor and its progenitor cells (219). ADSCs exhibit selective tumor homing ability, rendering them a suitable vehicle for anti-cancer drug delivery (143). By improving the targeting ability of drugs, it is also possible to improve the treatment efficiency and safety of high-dose use (220). ADSCs are often used in regenerative medicine and autologous transplantation, and have great potential for tissue regeneration and wound repair. In recent years, with the continuous development of nanotechnology, ADSCs-Exo have attracted increasing attention. ADSCs-Exo possess many therapeutic bioactive factors unique to stem cells, which can accelerate wound healing and are essential for tissue repair (221). They also play a key role in enhancing cell regeneration (222), promoting angiogenesis (223), regulating inflammation, and remodeling the extracellular matrix. ADSCs-Exo are often used in tendon repair, corneal skin regeneration, treatment of diabetic skin injury, regulation of inflammation and angiogenesis, fracture healing, etc (224).

ADSCs-Exo are an ideal potential drug delivery carrier with broad application prospects in tumor therapy (225). Lou et al. reported that ADSCs-Exo can be used as an effective carrier for the delivery of miR-199a. They can effectively improve the sensitivity of HCC cells to doxorubicin by targeting the mechanistic target of rapamycin kinase (mTOR) pathway (226). Studies have also shown that the delivery of miR-122 through ADSCs-Exo provides a new idea for improving the sensitivity of HCC to chemotherapy (227). Rezaeian et al. showed that ADSCs-Exo could affect prostate cancer, bladder cancer, and renal cancer cell lines. The 5637 cell line of primary bladder tumor, ACHN cell line of metastatic renal adenocarcinoma, LNCaP cell line of metastatic prostate cancer, and the prostate adenocarcinoma PC3 cell line were used. It was found that ADSCs-Exo exert a synergistic apoptotic effect on LNCaP, PC3, and 5637 cells, but not on ACHN cells. This difference was attributed to the increase in tumor protein 53 (TP53) expression and decrease in BCL2 gene expression in the PC3, 5637, and LNCaP cancer cell lines treated with exosomes (228). Liu et al. used ADSCs-Exo as a vector to deliver tumor suppressor miR-138–5p for the treatment of bladder cancer. The results showed that ADSC-Exo-miR-138–5p could inhibit the proliferation, migration, and invasion of bladder cancer cells *in vitro* and *in vivo*, This evidence indicated that ADSCs-Exo-miR-138–5p is a promising therapeutic agent for bladder cancer (213). Shojaei et al. used ADSCs-Exo to deliver the tumor suppressor miR-218 (downregulation was associated with EMT and angiogenesis) to breast cancer cells; the purpose of that study was to evaluate the tumor suppressor properties of miR-218 *in vitro*. The results demonstrated that ADSCs-Exo could effectively restore the levels of miR-218 in breast cancer cells and significantly reduce the expression of miR-218 target genes (RUNX family transcription factor 2 [RUNX2] and RPTOR independent companion of MTOR complex 2 [RICTOR]) in breast cancer cells (MDA-MB-231). The findings also indicate that miR-218 can prevent breast cancer progression by simultaneously targeting angiogenesis and EMT (229). Shojaei et al. also studied the usefulness of ADSCs-Exo as a carrier of miRNA-381. The treatment significantly downregulated the expression of genes and proteins related to EMT and inhibited the progression of triple-negative breast cancer *in vitro (* 230).

MSCs are the most widely used cells in cell therapy (231). Importantly, cell therapy is also associated with some significant risks. In some cases, ADSCs-Exo may also promote tumor progression. For example, Qu et al. found that ADSCs-Exo secreted into ascites activated the mitogen-activated protein kinase (MAPK) signaling pathway through forkhead box M1 (FOXM1) thereby regulating the ADSCs-Exo-mediated progression of ovarian cancer and promoting peritoneal metastasis of epithelial ovarian cancer (232). Wang et al. reported that ADSCs-Exo could promote the invasion, migration, and proliferation of osteosarcoma cells, as well as increase the expression of collagen β(1-O)galactosyltransferase 2 (COLGALT2) (233). Lin et al. also reported that ADSCs-Exo could activate the Wnt/β-catenin signaling pathway, thus promoting the migration and proliferation of breast cancer cell line MCF7 (234) (**Figure 4**).

### 2.4 Other MSCs derived exosomes

In addition to mesenchymal stem cells (MSCs) derived from umbilical cord, bone marrow and adipose tissue, exosomes derived from dental pulp, peripheral blood and placenta have great potential in disease treatment and biomarker diagnosis, and are expected to become a reserve force for treatment and diagnosis (235, 236). Dental pulp stem cell-derived exosomes (DPSC-Exos) have similar biological characteristics with bone marrow blasts and are closely related to tissue regeneration (237). Qiao et al. found that DPSC-Exos can inhibit periodontitis and promote epithelial healing in rats with periodontitis, and its mechanism is to regulate inflammation by inhibiting the IL-6/JAK2/STAT3 signaling pathway (238). The advantages of clinical transfusion and easy availability of peripheral-blood-derived exosomes have broadened the scope of their clinical application. Kang et al. prepared a mouse model of myocardial infarction and found that exosomes loaded with miR-21 mimics enhanced fibrosis, while exosomes loaded with miR-21 inhibitors reduced fibrosis, Human peripheral blood-derived exosomes loaded with miRNA can be used as a therapeutic tool for heart diseases (239). Placental mesenchymal stem cell-derived exosomes (Pd-MSC-Exos) can be detected in maternal blood as early as 6 weeks after conception (240), and their levels increase with gestational age (241). Zheng et al. analyzed the mechanism of Pd-MSC-EVs affecting liver fibrosis and found that Pd-MSC-EVs may inhibit the activation of hepatic stellate cells (HSC) through the miR-378c/SKP2 pathway. Thus, Pd-MSC-EVs are expected to become effective drug candidates for the treatment of liver fibrosis (242).

### 2.5 MSCs-derived exosomes for skin injury treatment

Mesenchymal stem cells are currently known to be the only cells that can be prepared on a large scale and have the ability to prepare exosomes on a large scale (243). Therefore, compared with exosomes from other sources, exosomes derived from mesenchymal stem cells have the following advantages: easy access, low immunogenicity, inhibition of the function of various immune effector cell types, promotion of immune regulation, anti-inflammatory, anti-aging and wound healing (191). Therefore, mesenchymal stem cells have become an ideal cell source in regenerative medicine and immunotherapy (244). MSCs-Exo can also be used for the treatment of skin lesions caused by chronic diabetes, Yang et al. combined hucMSCs-Exo and Pluronic F-127 (PF-127) hydrogel in diabetic rats and found that it could significantly accelerate the speed of wound healing, promote granulation tissue regeneration by increasing the expression of Ki67 and CD31. The expression of vascular endothelial growth factor (VEGF) and transforming growth factor beta-1 (TGFβ-1) was up-regulated to promote wound healing (245). Song et al. constructed ECM hydrogel loaded with ADSCs-Exo, and once injected into the wound site, ECM-Exo formed the hydrogel at a physiological temperature of nearly 37°C. ADSCs-Exo can be released slowly and continuously from the hydrogel to maintain a high concentration at the wound site. The ECM hydrogel gradually degrades *in vivo*. It can effectively reduce inflammation and promote angiogenesis, collagen deposition, cell proliferation and migration to accelerate wound healing (246), Wang et al. mixed collagen (COL-I) and platelet-rich plasma (PRP) and added thrombin to prepare a biological carrier, and delivered ADSCs-Exo in the carrier. The study found that the scaffold released a large number of growth factors, such as TGF-β, PDGF, FGF, HGF, and VEGF. These growth factors play a key role in wound healing and angiogenesis. Meanwhile, ADSCs-Exo also plays an important role in promoting tissue repair, regeneration and angiogenesis. *In vitro* experiments proved that the ADSCs- Exo based stents can induce angiogenesis, accelerate the healing process (247). The application of MSCs-Exo and its carrier provides a new idea for the treatment of skin injury. In addition, Hu et al. compared human amniotic mesenchymal stem cells (hAMSCs) and Schwann cell-like cells (SCLCs) derived exosomes in the treatment of peripheral nerve injury (PNI). The results showed that SCLCs-Exo enhanced the recovery of motor function in the rat model, alleviated gastrocnemial-muscle atrophy, promoted axon regeneration, myelination and angiogenesis, and up-regulated the expression of glial cell-derived neurotrophic factor, myelin positive regulator and myelin protein in Schwann cells. SCLCs-Exo is a potential new treatment for PNI (248).

## 3 Immune cell-derived exosomes (IM-Exo) in cancer therapy

Immune cells include NK cells, monocytes, macrophages, and granulocytes (mainly neutrophils) related to the innate immune response, as well as T lymphocytes, B lymphocytes, and DCs related to the adaptive immune response (249). These immune cells are involved in immune defense, immune surveillance, and immune clearance, playing an important role in maintaining human homeostasis.

The composition of IM-Exo consists of specific proteins, particularly tetrastransmembrane proteins (e.g., CD9, CD63, CD81, CD82), interacting with other proteins expressed on target cells (e.g., major histocompatibility complex [MHC] molecules, integrins) (250). In addition to proteins, unique lipids such as nucleic acid components include DNA, RNA (miRNA), lncRNA (metastasis associated lung adenocarcinoma transcript 1 [MALAT1], linc-POU class 3 homeobox 3 [linc-POU3F3], ZNFX1 antisense RNA 1 [ZFAS1], and growth arrest specific 5 [GAS5]). IM-Exo can stimulate immune cells (e.g., DCs, T cells) to fight pathogens, viral infections, and cancer cells. The effects of IM-Exo have become the focus of various nano-biomedicine studies, ranging from the medical use of diagnostic reagents based on nanoplatforms to the development of therapeutic interventions as well as vaccine applications. Thus, IM-Exo may be ideal for “immunotherapeutic diagnostics” (251).With the wide application of nanotherapeutics, IM-Exo have attracted considerable attention. IM-Exo possess immunomodulatory properties (252). They express various antigens on their surface and can be used for antigen presentation, immune activation, and metabolic regulation. Moreover, they can mediate crosstalk between innate and adaptive immunity (253). They can also reshape the pro-inflammatory microenvironment to inhibit tumor progression, or assist in the preparation of vaccines with anti-tumor effects (78). Additionally, they can promote tumor progression by inhibiting the killing effect of NK cells, CD8^+^ T cells, and other cells, promoting tumor cells, or inhibiting immune cells. Owing to their excellent biocompatibility, low immunogenicity, high loading capacity and easy cellular uptake, exosomes derived from immune cells are used as drug carriers in anti-tumor therapy to deliver miRNA, mRNA, or chemotherapy drugs (254, 255). Based on their favorable features, such exosomes have great potential in the treatment of diseases. In the section below, we will introduce the exosomes derived from T lymphocytes, B lymphocytes, DCs, macrophages, NK cells, neutrophils, and mast cells, as well as discuss their clinical advantages.

The current methods for the isolation of exosomes include differential centrifugation, immunoaffinity capture, exosome precipitation, and filtration membrane method (256). Differential centrifugation uses multiple cycles with different centrifugal forces and centrifugation times to achieve the effect of separation, and its essence is to separate exosomes according to the density and size difference between exosomes and other components in cells, large vesicles, and debris samples. It is the most commonly used method in the process of exosome isolation, also known as the “gold standard” (257). This method requires almost no technical expertise, is easy to operate, and only requires an ultracentruge for long-term operation, with less capital consumption. However, the disadvantage of this method is that it is time-consuming, requires a large number of starting samples, and has low efficiency when separating exosomes from viscous liquid (258). Immunoaffinity capture method, bound to specific antibodies that recognize exosome-specific surface markers. This method can separate the subsets of exosomes with high purity, and is often used for the isolation of plasma exosomes. The disadvantages are small sample volume and high reagent cost (259). Isolation of T-cell-derived exosomes, captured by anti-CD3 antibodies, Aneta Zebrowska et al. isolated CD3+ exosomes from human plasma and demonstrated their use as “T cell biopsies “ (260). Exosome precipitation methods include polyethylene glycol precipitation (PEG) and lectin precipitation. The advantages of this method are that the process has minimal harmful effect on the isolated exosomes, and the isolation method is fast and simple, and does not require technical expertise or expensive equipment (261). The disadvantages are limited analytical power and lack of selectivity. A filter membrane was used to separate exosomes from other macromolecules. It does not require special instruments and takes a short time, but it will cause deformation and rupture of the exosomes, which will affect the results of the analysis (258). However, this situation can be reduced by monitoring and regulating the transmembrane pressure. Exosome characterization is the quantitative and qualitative analysis of the total number of exosomes, proteins, lipids and DNA/RNA using physical and chemical composition analysis (262). Physical analysis is achieved by nanoparticle tracking analysis (NTA), dynamic light scattering (DLS), flow cytometry, transmission electron microscopy (TEM), and resistive pulse sensing (RPS), which provides insight into particle size and or concentration. Chemical composition analysis is usually performed by staining, immunoblotting, or proteomic analysis and gives information about the content of the isolated vesicles (263).

Luana Lugini et al. separated mononuclear cells (PBMC) from whole blood after Ficoll-Histopaque 1077 gradient, then added monoclonal antibodies against CD3, CD4, CD8, CD20 and CD14, and purified NK cells by negative magnetic bead selection. After purification, the selected NK cells were CD56+,CD3-,CD14-, and the supernatant was collected. The exosomes were isolated from the supernatant of NK cells by using an ultracentrifuge (264). Due to the scarcity of NK cells in human lymphocytes, changes in phenotype, and impaired function during cancer progression, it is necessary to develop new protocols to activate and expand NK cells to achieve adoptive transfer in sufficient numbers *in vitro* and to make them a viable approach to control the immune system against cancer (265), Subsequently, many candidate effectors were identified based on proteomic analysis and functional studies. Such as Fas ligand, TRAIL, NKG2D, beta actin and fibrinogen, that NK cells derived EVs may be as a viable cancer immunotherapy strategies (265).

Aled Clayton et al. studied expression and function on the exosomes of antigen presenting cells (APC). It was found that both CD55 and CD59 are expressed by APC-derived exosomes and play a role in protecting them from complement attack (266). The specific mechanism is that CD55 can inhibit the initial deposition of complement C3b, and CD59 can inhibit the formation of membrane attack complex. To play a protective role. Veerman et al. compared EVs obtained from conditioned cell culture medium and 250μl or 3 ml plasma by five commonly used methods based on different principles, including precipitation, membrane affinity, size exclusion chromatography, iodixanol gradient, and phosphatidylserine affinity, and found that EV subsets and lipoproteins are highly heterogeneous in different isolation methods. The precipitation method has the smallest concentration of EV, the membrane affinity method has a large cup type of EV, and the size exclusion chromatography method has the highest heterogeneity of EV population. The methods used for the separation of different samples are different, so the appropriate method should be adopted in the separation (267). Therefore, in the process of sample processing and separation, samples from different sources are separated by different methods. It is very important to choose the appropriate method according to the characteristics of the separated samples.

As drug nanocellulars, exosomes derived from immune cells have good biocompatibility, low immunogenicity, high stability and inherent tumor targeting, which can be used for tumor targeted therapy and is expected to be used in clinical practice as a cancer vaccine (268). Krug et al. investigated whether the use of combined isolation of exosomal RNA (Exo-RNA) and cell-free DNA (cfDNA) could improve blood liquid biopsy for EGFR mutation detection in NSCLC patients. Matched pretreatment tumor and plasma were collected from 84 patients, and it was found that Exo-RNA based liquid biopsy improved the sensitivity of liquid biopsy, and can be used in any cancer patient suitable for liquid biopsy, which has great potential in future research (269). In addition, Bernard et al. reported for the first time the feasibility of DC-derived exosome (DEX) vaccine in phase I clinical trials of melanoma patients and the safety of exosome administration (270). In this trial, 15 patients with stage IIIB and IV melanoma were recruited and received 4 doses of exosome vaccine. Two weeks after the fourth vaccination, MHC class II molecules, peptides, and tumor status were detected, and mild inflammatory reaction was found at the vaccine site, without exogenous hypersensitivity, which could activate and recruit T cells to the tumor area (270), resulting in tumor reduction. Morse et al. also studied the safety, feasibility and effectiveness of DEX loaded with MAGE tumor antigen in NSCLC patients (271), which proved that DEX could be used in clinical research. With the progress of phase II and phase III studies, DEX is expected to become a new immunological method for tumor treatment.

### 3.1 DC-derived exosomes (DEX)

DCs are antigen-presenting cells (APCs) with the unique ability to induce primary and secondary immune responses. They also play an important role in tumor immunotherapy, and are involved in anti-tumor immunity, activating tumor-specific T cells to eliminate tumor cells. DCs are often used in the preparation of vaccines (272, 273); however, their use is limited due to the high manufacturing cost, time constraint, difficult preservation of living cells, and possible functional and phenotypic changes after injection (274). DEX have also attracted attention as immune cell-derived exosomes. They are characterized by the expression of tumor antigens, MHC class I (MHC-I), class II (MHC-II), and T cell costimulatory molecules on their surface. After capturing and internalizing the antigen-MHC complex, these antigen-MHC complexes are presented to T cells via APC, thereby triggering the release of antigen-specific CD4^+^ and CD8^+^ T cells (156). DEX are nanovesicles containing functional MHC-peptide complexes that promote T cell-dependent tumor rejection. There are three mechanisms through which DEX stimulate T cell production. Firstly, DEX directly stimulate T cells to exert their effect; however, direct T cell stimulation appears to be inefficient in priming naive T cells. Secondly, the antigenic peptide-MHC complex is transferred to the APC for more efficient stimulation of T cell responses by APC presentation. Thirdly, T cells may be indirectly activated through tumorigenesis (275). Through research on mice, Viaud et al. also found that DEX could promote the proliferation and activation of NK cells by promoting IL-15Rα and NKG2D, thereby producing an anti-metastatic effect mediated by NK1.1 cells (276). DEX carry numerous molecules related to the immune function of DCs; these molecules are bound by tumor cells, transforming them into immunogenic targets. Compared with exosomes from immature DCs, those derived from mature DCs have less loss after endocytosis and greater ability to stimulate T cells (277, 278). These observations provide a good basis for targeted therapy of tumors. DEX can be used to load neoantigens, which is not susceptible to environmental influences and can retain function and phenotype as a new nanovaccine, which can more easily transport antigens to lymph nodes and trigger a strong immune response (268, 274). Lu et al. studied exosomes derived from HCC antigen-expressing DCs in three different HCC mouse models. They demonstrated that α-fetoprotein-rich DEX could trigger effective antigen-specific anti-tumor immune responses and reshape the tumor microenvironment (TME) in HCC mice, thus providing a cell-free vaccine option for HCC immunotherapy (279). Zhong et al. used microwave ablation combined with DEX to treat mice with HCC. They found that the number of CD8^+^T cells at the tumor site and the plasma IFN-γ concentration were increased, whereas the number of regulatory T cells and the IL-10 concentration were decreased. The results showed that the combination of microwave ablation with DEX can significantly inhibit tumor growth and improve the immune microenvironment, thereby providing a new direction for the development of vaccines based on DCs and DEX (280). The membrane structure of DEX avoids high degradation, while ensuring good biocompatibility and *in vivo* safety. Compared with DC-based vaccines, DEX have higher immunogenicity and stronger resistance to immunosuppression, and have shown better anti-tumor effects in preclinical studies. Hao et al. reported that intravenous injection of an exosome vaccine is superior to subcutaneous injection, inducing stronger anti-tumor immunity. A Phase I study of DEX failed to demonstrate its immune competence (281). Therefore, Viaud et al. developed second-generation DEX with enhanced immunostimulatory properties. The clinical grade process of the IFN-γ-DEX vaccine and its quality control parameters currently used in phase II trials were studied. IFN-γ is a key cytokine that regulates the expression of CD40, CD80, CD86, and CD54 induced by DCs on DEX, leading to direct and effective peptide-dependent CD8^+^T cell weight gain potential *in vitro* and *in vivo (* 282).

Zhu et al. developed an anti-tumor vaccine candidate by coupling mucin 1 (MUC1) glycopeptide antigen to DEX. They found that MUC1-DEX induced high MUC1-specific immunoglobulin G antibody titers with strong binding affinity to MUC1-positive tumor cells *in vivo*. This treatment enhanced cytotoxicity of CD8^+^T cells from immunized mice against MUC1-positive tumor cells. It also inhibited tumor growth and prolonged the survival time of mice in preventive and therapeutic tumor-bearing mouse models (283).

Silva et al. reported that DEX play a role in tissue regeneration. DEX are naturally loaded with chemoattractants, which can promote cell recruitment. Osteopontin and MMP9 have been confirmed in EVs (284). Triptolide has beneficial effects in the treatment of cancer (e.g., gastric carcinoma, lung cancer), but causes multi-organ toxicity. DCs are the main targets of triptolide, inducing immunosuppression. Rao et al. packaged triptolide in DEX for targeted delivery to reduce toxicity. They reported that triptolide with DEX could play a role by reducing CD4^+^ T cells and increasing regulatory T cells *in vivo* to reshape the immune environment (285).

**Figure 5:**
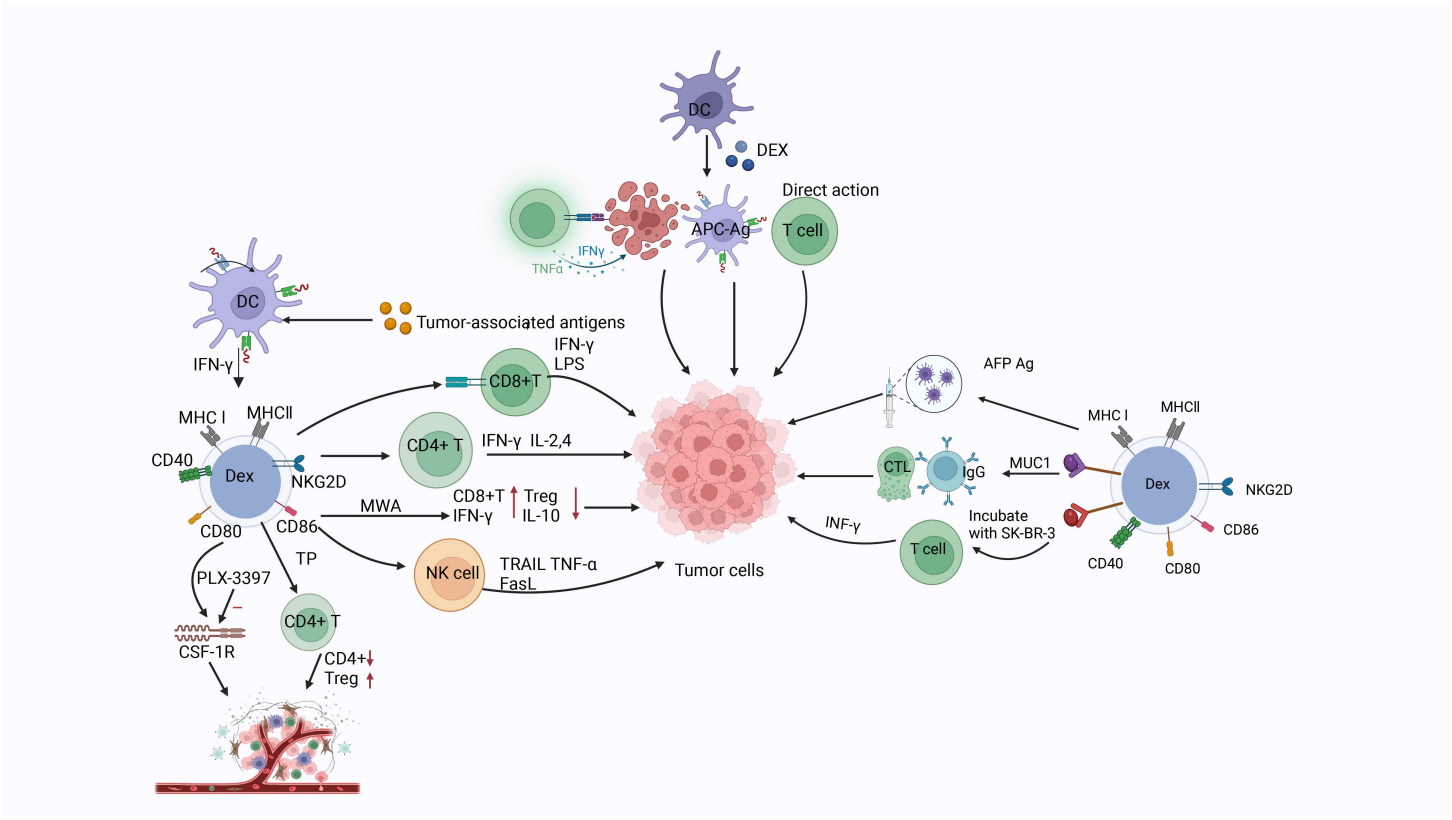
The related markers of dendritic cell-derived exosomes, the three mechanisms by which Dex functions (direct action, indirect action through secretion-related factors, and action through T cells), as well as the mechanisms and relevant targets of dendritic cell-derived exosomes on tumor cells.

Barnwal et al. reported that myeloid-derived DEX were obtained from bone marrow in the presence of tumor antigen. Studies have demonstrated that colony-stimulating factor 1 receptor (CSF-1R) inhibitor (PLX-3397) targeting the colony-stimulating factor 1/CSF1R (CSF1/CSF1R) signaling pathway can deplete tumor-associated macrophages (TAMs) and myeloid-derived suppressor cells responsible for an immunosuppressive TME. In a B16-F10 mouse model of melanoma, DEX combined with PLX-3397 regulated the TME by transferring Th1/Th2 to dominant Th1 population and depleting TAMs and myeloid-derived suppressor cells. These findings also provide a new strategy for the treatment of melanoma (286). DEX carry many molecules associated with the immune function of DCs, and their incorporation into tumor cells can transform them into immunogenic targets. Romagnoli et al. treated breast cancer cell line SK-BR-3 with DEX, and subsequently used these DEX to stimulate SK-BR-3 cells sensitized with CD3^+^ T cells. The investigators generated DEX-SK-BR-3-trimer CD3^+^ T cells, and revealed that the sensitizing T cells cultured from tumor cells treated with DEX had a stronger ability to secrete IFN-γ compared with non-DEX-treated cells. These data suggest that incorporation of DEX into tumor cells enhances the activation of T cells, thus potentially producing a more effective response. Collectively, these findings imply that DEX may become an important tool in cancer immunotherapy (287) (**Figure 5**).

### 3.2 Macrophage-derived exosomes

Macrophages develop from hematopoietic stem cells, namely monocytes in the bone marrow. They play roles in phagocytosis (a major mechanisms of innate immunity) and antigen presentation. Five activated macrophage phenotypes have been identified, namely M1 macrophages, M2 macrophages, CD169^+^ macrophages, TCR^+^ macrophages, and TAMs; inactivated macrophages (termed M0) have also been identified. Among these phenotypes, M1 and M2 have been primarily studied (201, 288). These two phenotypes were distinguished according to their function and the level of inflammatory factor secretion. M1 macrophages exhibit an anti-tumor and pro-inflammatory phenotype, and can release pro-inflammatory cytokines, such as tumor necrosis factor-α (TNF-α), C-C motif chemokine ligand 2 (CCL2), IL-6, inducible nitric oxide synthase (iNOS), IL-1α, IL-1β, IL-12, IL-23, IL-18, type I IFN (-α and -β), C-X-C motif chemokine ligand 1–3 (CXCL1–3), CXCL5, and CXCL8–10. In contrast, M2 macrophages show a pro-tumor and anti-inflammatory profile (289). Arabpour et al. reported that MSC-Exo reduced inflammation by promoting M1 to M2 polarization and increasing anti-inflammatory cytokines and chemokines (290). Pritchard et al. reported that lung tumor-derived exosomes can also promote the polarization of M2 macrophages (290). Inactivated M0 can be induced to M1 under the action of lipopolysaccharide, TNF-α, and IFN-γ, while cytokines (e.g., IL-4 and IL-13) are required to induce M0 to M2 (291, 292). M1 macrophage exosomes have the ability to target lymph nodes and can be absorbed by local macrophages and DCs. Macrophage-derived exosomes are involved in immune activation and regulation, and serve as anti-cancer drug carriers.

Rayamajhi et al. designed hybrid exosomes by hybridizing small EVs from mouse macrophages with synthetic liposomes. The hybrid exosomes were loaded with water-soluble doxorubicin. The toxicity of the exosome-doxorubicin hybrid to cancer cells and drug release were enhanced under acidic conditions; this finding indicates the possibility for drug delivery to the acidic cancer environment (293). Li et al. developed a macrophage-derived exosome-coated polylactic acid-glycolic acid nanoplatform for targeted chemotherapy of triple-negative breast cancer. To further improve tumor targeting, the surface of exosomes was modified with peptides (253). The results showed that the engineered exosome-coated nanoparticles significantly improved the cellular uptake efficiency of doxorubicin and anti-tumor effect *in vivo* and *in vitro*, and induced the apoptosis of tumor cells.

**Figure 6:**
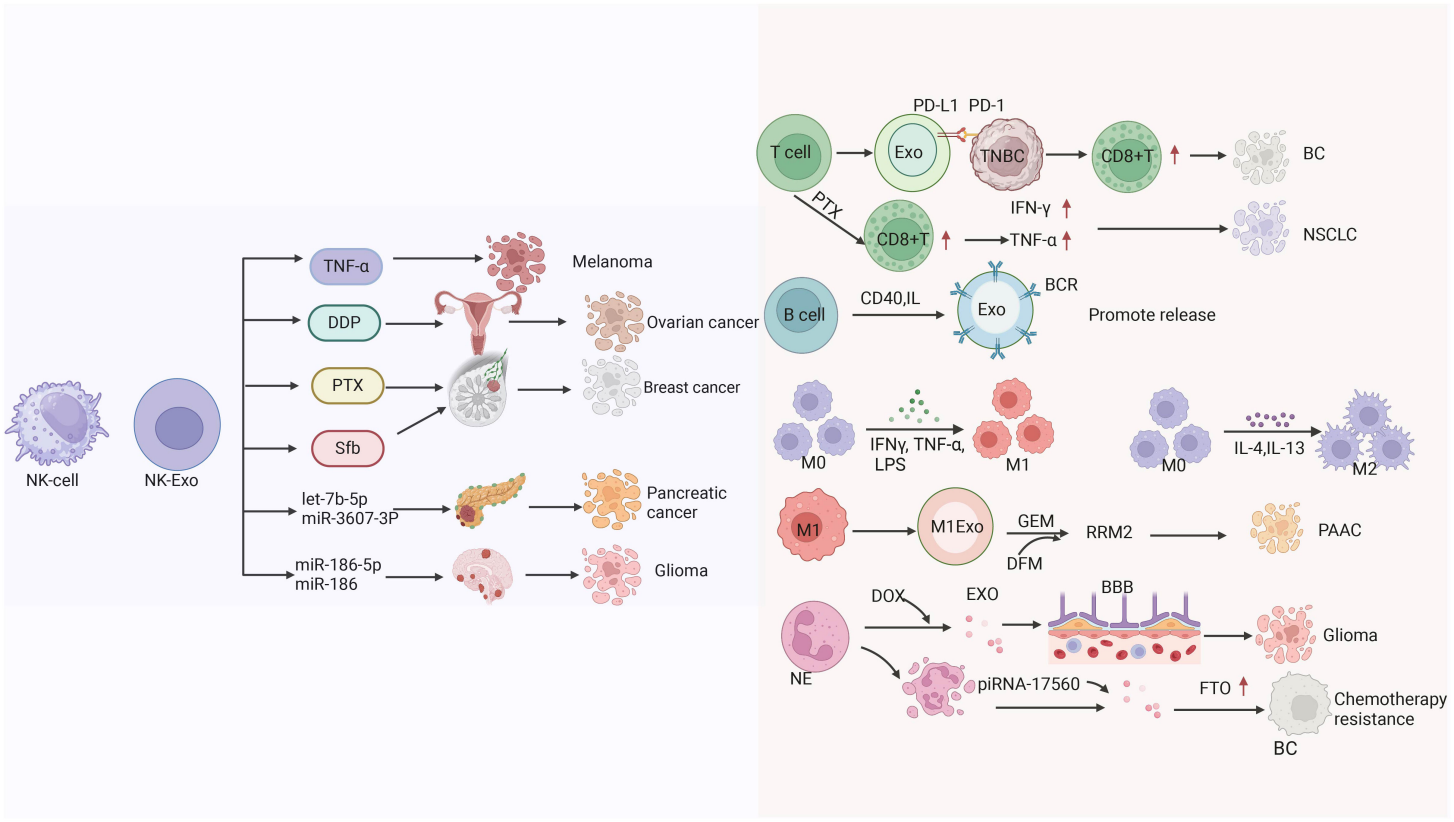
The mechanisms by which extracellular vesicles derived from natural killer cells transport different substances (TNF-α, Cisplatin (DDP), paclitaxel (PTX), Sorafenib (Sfb), the miRNA) to kill tumors. as well as the mechanisms of extracellular vesicle therapy derived from T cells for BC and NSCLC treatment, and the related mechanisms of extracellular vesicles derived from B cells, macrophages, and neutrophils in killing tumors.

Despite the availability of many options for the treatment of pancreatic cancer, chemotherapy is currently the main therapeutic modality (294). Chemotherapy drugs play a major role in the treatment of cancer; however, the development of chemotherapy resistance limits its efficacy. Therefore, it is necessary to develop more effective treatments. Zhao et al. have shown that it is possible to develop a specific M1 macrophage-derived exosome-gemcitabine delivery system and load it with noracilor (DFX). DFX is designed to deplete iron, thereby inhibiting the expression of the ribonucleotide reductase regulatory subunit M2 (RRM2), This approach improved the efficacy of gemcitabine. This delivery system can inhibit tumor cell proliferation, attachment, and migration, reverse the chemoresistance of tumor cells to gemcitabine, and significantly enhance the efficacy of gemcitabine. Therefore, this system provides a new strategy for the treatment of pancreatic cancer (295) (**Figure 6**).

### 3.3 Neutrophil-derived exosomes

Neutrophils are the most abundant type of innate immune cells in the human body with a tendency to inflammation (296). Neutrophil-derived exosomes have the same effect to achieve tumor targeting. Surgical resection is commonly used for the treatment of glioma. However, surgery is often accompanied by infection and metastasis, while chemotherapy after surgical resection has a poor effect due to the existence of the BBB. Wang et al. developed a new pro-inflammatory drug delivery system to overcome the risk of inflammation and metastasis after surgical resection of glioma using neutrophil-derived exosomes as a carrier of doxorubicin. Through *in vivo* and *in vitro* experiments, it was confirmed that neutrophil-derived exosomes had inflammatory tendency and could target the inflammatory site of tumor to improve the therapeutic effect and the overall survival of patients with glioma (297). Neutrophil-derived exosomes can also act as carriers by delivering cytotoxic proteins and activating caspase signaling pathways (298). Vargas et al. found that neutrophil-derived exosomes could be internalized by airway smooth muscle and alter its proliferative properties. These exosomes play an important role in asthma progression, promoting airway remodeling in patients with severe and corticosteroid-insensitive asthma (299). Ou et al. found that senescent neutrophil-derived exosome piRNA-17560 enhanced cellulite and obesity-associated protein (FTO) expression in breast cancer cells, as well as chemotherapy resistance. Senescent neutrophils may be a therapeutic target for breast cancer (300). Tyagi et al. study examined exosomal miR-4466 from N2 neutrophils in smokers and non-smokers. They observed that the expression of exosomal miR-4466 from neutrophils was increased in smokers versus non-smokers. Therefore, neutrophil-derived exosomal miR-4466 can be used as a promising predictor of metastatic disease in smokers (301) (**Figure 6**).

### 3.4 T lymphocytes-derived exosomes

T lymphocytes, thymus-dependent lymphocytes, are pluripotent stem cells derived from bone marrow (302). These cells are an integral part of adaptive immunity. T cells have different subsets with varied functions, and can play a role through direct contact between cells or the transfer of secreted molecules (303). According to the phenotype, T cells can be mainly divided into CD4^+^ Th cells, CD8^+^ cytotoxic T cells, follicular helper T cells, and regulatory T cells. Following maturation in the thymus, T cells migrate to peripheral tissues (304).

A growing number of studies have shown that immune cells participate in cell communication by secreting exosomes (305). Among immune cell-derived exosomes, those derived from T cells participate in the anti-tumor effect of cancer immunotherapy by mimicking the effect of parental cells (303). T cells produce exosomes that reflect their characteristics, such as direct killing of target cells, regulating B cells to produce antibodies, and producing cytokines (e.g., IL-7, IL-10, IL-12, IL-17, INF-γ) (306), thus creating the optimal microenvironment for paracrine and autocrine immune cells. T cell-derived exosomes can also play an important role in intercellular signal transduction and activate other immune cells, thereby participating in the corresponding immune regulation process (303).

Programmed cell death 1 (PD-1) is widely expressed in tumor-infiltrating lymphocytes in triple-negative breast cancer, and cell-surface PD-1 transduces negative signals for effector T cell activity during cell-cell contact (307). PD-1 is secreted in the form of exosomes from activated T cells and can remotely interact with cell-surface or exosomal programmed death ligand 1 (PD-L1). This interaction restores tumor surveillance by attenuating PD-L1-induced suppression of tumor-specific cytotoxic T cell activity and exosome PD-1 anti-PD-L1 function. Overall, it enhances the activity of cytotoxic T cells (308).

Paclitaxel (PTX) is a chemotherapeutic drug with limited use due to its systemic toxicity. Chimeric antigen receptor-T (CAR-T) cell-derived exosomes (CAR-T-Exo) contain tumor-targeted CAR and cytotoxic particles (granzyme B [GZMB] and perforin [PRF]), which can be used in the treatment of tumors and are considered potential carriers of paclitaxel (268). Zheng et al. reported that CAR-T-derived exosomes can deliver paclitaxel, reprogram the TME, and reverse immunosuppression to increase the levels of CD8^+^T cells, IFN-γ, and TNF-α, thereby enabling the treatment of non-small cell lung cancer (309) (**Figure 6**).

Huang et al. performed omics analysis of CD4+T cell-derived exosomes from patients with rheumatoid arthritis (RA) and found that the expression of dihydropyrimidinase associated protein 3 (DPYSL3) was significantly up-regulated and the expression of proteasome activating complex subunit 1 (PSME1) was significantly down-regulated. These differentially expressed genes may be involved in the pathogenesis of RA, thus DPYSL3 and PSME1 are expected to be biomarkers for RA diagnosis (310). Xu et al. found that miR-186–5p in CD8 T cell-derived exosomes caused renal inflammation and tissue damage. miR-186–5p directly activates TLR7/8 signaling axis in renal tubules to cause renal inflammation, which reveals the specific pathogenic mechanism and reason of the pathogenic role in T cell-mediated renal dysfunction and provides new ideas for the treatment of nephropathy (311). T cell-derived exosomes are still in the exploratory stage, and continuous efforts are still needed to achieve clinical transformation. We believe that with the continuous efforts of scholars, T cell-derived exosomes can become a powerful tool for disease treatment.

### 3.5 B lymphocyte-derived exosomes

B lymphocytes are bone marrow-dependent lymphocytes. Mature B cells migrate out of the peripheral blood, and enter the spleen and lymph nodes. Following stimulation by antigens, they proliferate and differentiate into plasma cells, and participate in humoral immunity. B cells differentiate into effector cells with the synthesis of exosomes, which is initiated upon stimulation by activation signals, in particular T cell “help” via CD40 and IL-4 signaling. B cell-derived exosomes induce antigen-specific, MHC-II-restricted T cell responses, suggesting a role for exosomes in antigen presentation *in vivo (* 312). B cell-derived exosomes also contain immunoglobulins that deliver surface B cell receptor-bound antigens into the endosomal/exosomal pathway (313). Saunderson et al. demonstrated that primary B cells release high levels of exosomes in response to CD40 and IL-4 signaling. The absolute number of splenic immune cell subsets was determined to investigate the immune cells that respond to Ag of B cell-derived exosomes. After immunization, the number of NK cells, B cells, CD4 T cells, and CD8 T cells in the spleen was significantly increased (314) (**Figure 6**). Dan Ma et al. characterized B lymphocyte-derived exosomes in fatal Pneumocystis pneumonia (PCP) and found significant alterations in histone H1.3, vimentin, and tyrosine protein phosphatase non-receptor type 6 (PTPN6) levels. The proinflammatory effects of B-cell-derived exosomes from PCP on CD4+T cell responses were revealed. This finding provides a new idea for the study of PCP (315).

### 3.6 NK cell-derived exosomes (NK-Exo)

NK cells, which constitute a small population of cells, can kill target cells in a non-specific manner in the human body. This killing activity is innate, does not require prior antigen sensitization, and is not restricted by MHC. NK-Exo expressed various NK receptors/markers, including CD56, CD69, cytotoxic receptors (e.g., NKG2D), NKp44, NKp46, NKp30, CD40L, PD-1, and molecules involved in tumor cell recognition and immune synapse formation (lymphocyte function-associated antigen 1 [LFA-1], DNAM1). They also carry cytotoxic proteins (e.g., PRF, GZMA, GZMB, and Fas ligand [FasL]) and cytokines (e.g., IFN-γ and TNF-α) (316). NK-Exo recognize and kill cancer cells through various mechanisms *in vitro* and *in vivo*. They exert an anti-tumor effect due to the presence of PRF and FasL, which trigger the intrinsic pathway and promote the release of cytochrome-c. FasL triggers the extrinsic apoptotic pathway by activating caspase 8 (CASP8), CASP3, and poly(ADP-ribose) polymerase (PARP) (317). NK-Exo contain potent cytotoxic proteins that induce apoptosis in targeted cancer cells. Furthermore, EVs derived from cancer cells carrying NK ligands may evade immune surveillance and responses (318). Zhu et al. found that NK-Exo expressed two typical exosomal proteins (CD63 and ALG-2 interacting protein X [ALIX]) and two functional NK proteins (PRF and FasL) (317). Moreover, NK-Exo can secrete TNF-α, thereby affecting signaling pathways that control cell proliferation. NK-Exo exert a cytotoxic effect on melanoma cells *in vitro*, without significant side effects on normal NK-Exo cells (317).

Luo et al. found that NK-Exo could activate NK cells from the immunosuppressed TME. They also showed that cisplatin-loaded NK-Exo could enhance the sensitivity of drug-resistant ovarian cancer cells to cisplatin, thus playing an anti-proliferation role (319). Han et al. also reported that paclitaxel embedded in NK-Exo effectively inhibited the proliferation and induced apoptosis of breast cancer cells. Hashemi et al. constructed a drug delivery system combining NK-Exo and the anti-cancer drug sorafenib (NK-Exo-SFB), which exerted an inhibitory effect on breast cancer cells (320). Di Pace et al. analyzed miRNAs in NK-Exo, revealing that let-7b-5p was enriched in exosomes. The let-7b-5p belongs to the let-7 family of miRNAs with key tumor suppressor functions. It has an anti-proliferation effect on pancreatic cancer cells (321). Sun et al. also reported that miR-3607–3p of NK-Exo can inhibit the progression of pancreatic cancer (322). Notably, NK-Exo eliminated leukemia cells isolated from patients with acute and chronic leukemia and inhibited the growth of hematopoietic colonies; these findings led to the development of a cell-free therapy for leukemia (323). Wang et al. demonstrated that treatment with NK-Exo significantly inhibited TGF-β1-induced proliferation and activation of hepatic stellate cells, as well as liver fibrosis, thus providing a new means for the treatment of liver fibrosis (290). Neviani et al. reported that NK exosomes carry the tumor suppressor gene miR-186–5p, which impairs the growth of neuroblastoma cells *in vitro* and *in vivo (* 318). They demonstrated that NK-Exo carrying tumor suppressor gene miR-186p exhibited cytotoxicity against neuroblastoma cell lines (**Figure 6**).

## 4 Tumor cell-derived exosomes (TEX) in cancer therapy

Tumor-generated EVs, also known as TEX, contain tumor antigens and have been used as a specific stimulator of immune responses against tumors. TEX, as a means of “liquid tumor biopsy,” are considered a promising biomarker for the early detection of malignancies in humans. Moreover, they provide a promising method for monitoring cancer progression or response to treatment (324). TEX carry many molecules and factors derived from tumor cells. These exosomes are recognized and taken up by immune cells, playing an important role in communication between cancer cells and immune cells (325). TEX can inhibit the function of immune cells and help tumors escape immune surveillance in the TME (326). Li et al. conducted a study on engineered tumor-derived exosomes. They discovered that these exosomes could inhibit the cytotoxicity of NK cells by inhibiting the expression of activated receptors on NK cells, leading to immune escape (326). Zhu et al. studied exosomes derived from oral cancer and found enrichment of TGF-β1. The cytotoxicity of NK cells was weakened at 7 days after co-culture of exosomes derived from oral cancer and NK cells, and TGF-β1 inhibited the function of NK cells (327). Tumor-derived exosomes can transform an anti-tumor environment into a pro-tumor environment by inducing the differentiation of stromal cells into tumor-associated cells. Exosomes derived from tumor-associated stromal cells mutually trigger EMT of tumor cells, resulting in treatment resistance and metastasis (328).

TEX play an important role in tumor growth, metastasis, and immune regulation (329). Furthermore, they monitor the development of diseases and serve as a diagnostic marker. Wu et al. found that overexpression of calcyphosine 1 (CAPS1) by CRC cell-derived exosomes enhanced the migration of normal colonic epithelial FHC cells. Therefore, inhibition of tumor exosome secretion is a therapeutic option for patients with metastatic CRC. Wu et al. reported that exosomes derived from tumor cells can transfer specific lncRNAs to receptor cells that regulate the TME and promote angiogenesis. Invasive and migratory TEX lncRNAs have become new non-invasive tumor biomarkers for early diagnosis and evaluation of prognosis (330). Below, research progress on exosomes derived from different tumor cells is introduced, and the potential of TEX as a promising marker for cancer diagnosis is explained.

### 4.1 TEX of the digestive system

Liver cancer cell-derived exosomes, i.e., HCC-derived exosomes (HCC-Exo), have been shown to attenuate the cytotoxicity of T and NK cells and promote immunosuppressed M2 macrophages, N2 neutrophils, and regulatory B cells (329). Yu et al. reported that miR-21–5p from HCC-Exo directly targeted the UTR of Ras homolog family member B (RhoB) in human monocyte-derived leukemia (THP-1) cells and promoted TAM polarization. The evidence indicates that tumor-derived miR-21–5p promotes the malignant progression of HCC, thereby mediating intercellular crosstalk between tumor cells and macrophages. Targeting M2-like TAMs and blocking their associated signaling pathways may provide specific and novel therapeutic approaches to HCC treatment (331). Zhang et al. reported that HCC cells can secrete exosome circular ubiquitin-like PHD and ring finger domain 1 RNA (circUHRF1). The circRNA mainly acts as a miRNA sponge by binding to miRNA and subsequently promoting the expression of miRNA targeted genes. The circUHRF1 degrades miR-449c-5p, thereby upregulating T-cell immunoglobulin mucin family member 3 (TIM-3) expression, inhibiting NK cell-derived IFN-γ and TNF-α, and promoting immunosuppression. These findings provide a potential treatment strategy for patients with HCC (253).

Shen et al. tested the immunoregulatory effect of gastric cancer cell-derived exosomes (AGS-Exo) on MSCs. MSCs were stimulated with AGS-Exo, which led to abnormal activation of the nuclear factor-κB (NF-κB) signaling pathway. The effects of AGS-Exo-stimulated MSCs significantly attenuated the function of T cells and macrophages. Therefore, AGS-Exo affects the immunomodulatory function of MSCs through the NF-κB signaling pathway, thus enhancing the ability of MSCs to activate immune cells, maintain an inflammatory environment, and support tumor growth (332). High mobility group box 1 (HMGB1) is a non-histone chromatin-related protein widely distributed in eukaryotic cells. It is involved in DNA damage repair and maintenance of genome stability. HMGB1 can promote tumorigenesis, while it can also mediate immunogenic cell death during chemoradiotherapy and enhance anti-tumor immunity (333). Liu et al. studied the regulatory effect and potential mechanism of HMGB-1 in AGS-Exo on the polarization of M2-like macrophages. They revealed that HMGB-1 interacts with the transcription factor POU2F1 to inhibit the transcriptional activity of p50 and inactivate the NF-κB signaling pathway, thereby inducing the polarization of M2 macrophages (334).

**Figure 7:**
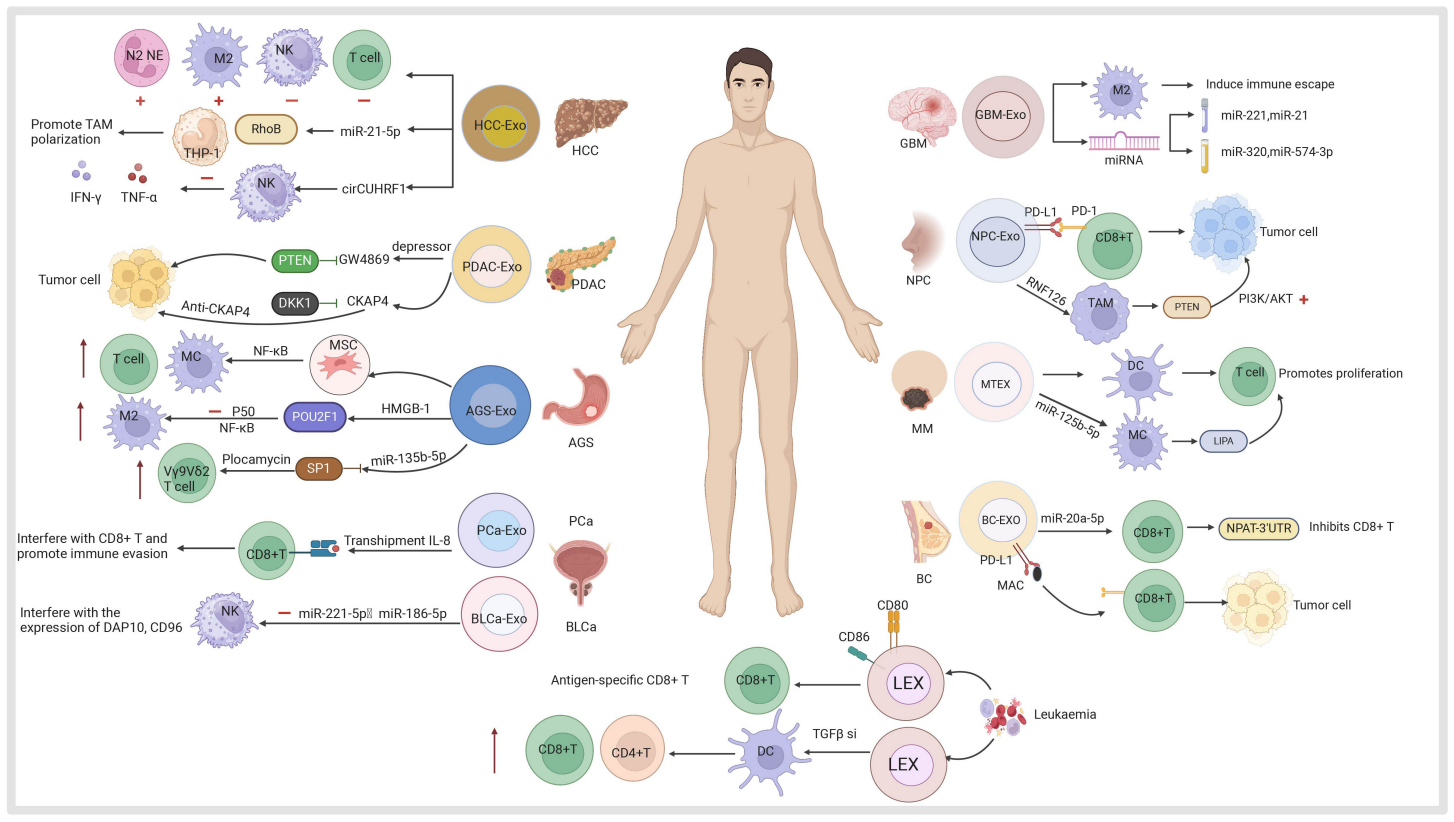
Exosomes derived from various tumor cells, including hepatocellular carcinoma (HCC), pancreatic ductal adenocarcinoma (PDAC), gastric cancer (AGS), bladder cancer (BLCa), prostate cancer (PCa), glioblastoma (GBM), nasopharyngeal carcinoma (NPC), melanoma (MM), breast cancer (BC) and leukemia are utilized for tumor diagnosis and targeting mechanisms in treatment.

Vγ9Vδ2 T cells, as a subtype of T cells, express T cell receptors composed of γ and δ chains and play an important role in innate and adaptive immune surveillance (335). AGS-Exo are effectively taken up by Vγ9Vδ2 T cells to induce cell apoptosis. This uptake also reduced the production of cytotoxic cytokines IFN-γ and TNF-α. Li et al. demonstrated that exosomal miR-135b-5p was successfully delivered to Vγ9Vδ2 T cells. Exosomal miR-135b-5p impairs the function of Vγ9Vδ2 T cells by targeting specific protein 1 (SP1) (336). SP1 inhibitor plicamycin was also administered to prevent SP1 function. These results suggested that AGS-Exo impaired the function of Vγ9Vδ2 T cells through the miR-135b-5p/SP1 pathway (336). Targeting the exosomal miR-135b-56/SP1 axis may improve the efficiency of immunotherapy for gastric cancer based on Vγ9Vδ2 T lymphocytes (**Figure 7**).

### 4.2 Pancreatic cancer cell-derived exosomes (PEX)

PEX is involved in drug resistance, immune evasion, metabolic reprogramming, and distant metastasis of pancreatic cancer, and plays a key role in the occurrence and development of this disease. Their extensive differential expression and functional content render PEX a promising screening tool and therapeutic target (337).

Exosomes secreted by cancer-associated fibroblasts in the TME promote tumor proliferation and chemotherapy resistance by inhibiting a tumor suppressor (PTEN) during the treatment of PDAC with gemcitabine. Therefore, exosome inhibitor GW4869 should be used to block the inhibition of PTEN *in vivo* and improve the anti-tumor effect of chemotherapy drugs (338). Exosomes secreted by PDAC can be used as diagnostic markers. The function of PDAC exosomes is mainly reflected in mediating immune escape, enhancing resistance to gemcitabine therapy, and promoting the progression of PDAC through the release of proteins and miRNAs. Exosomes produced by PDAC cells highly express cytoskeleton-associated protein 4 (CKAP4), a novel dickkopf WNT signaling pathway inhibitor 1 (DKK1) receptor. CKAP4 is highly expressed in patients with pancreatic cancer. Inhibitors of this molecule can prevent binding to DKK1, thereby inhibiting the proliferation and migration of PDAC cells. Hence, CKAP4 is a potential new target for the treatment of PDAC (339) (**Figure 7**).

### 4.3 Head and neck cancer cell-derived exosomes

Exosomes play a key role in brain tumors. Exosomes produced by glioblastoma enable cell-to-cell communication and promote glioblastoma progression. This occurs by inducing M2 macrophages around the glioblastoma to achieve immune escape. The expression of miRNAs was detected in the cerebrospinal fluid and plasma of patients with glioblastoma. Regardless of the role and activity of these miRNAs, they can be used as diagnostic markers. Specifically, miR-221 and miR-21 have been evaluated in the cerebrospinal fluid of patients with glioblastoma. Similarly, miR-320 and miR-574–3p in plasma can also be used as diagnostic biomarkers (340).

Yang et al. reported that nasopharyngeal carcinoma-derived exosomes (NPC-Exo) highly express PD-L1, which can bind to PD-1 on CD8^+^T cells. This leads to inhibition of CD8^+^T cell activity, promotion of nasopharyngeal carcinoma tumor growth in mice, and evasion of T cell immunity by nasopharyngeal carcinoma cells (341). Yu et al. found that NPC-Exo can help RNF126 (an E3 ubiquitylation ligase that acts as an oncogene) to enter TAMs and bind to and ubiquitate PTEN (a tumor suppressor termed tension homolog) (78). PTEN regulates tumor radiotherapy and chemotherapy resistance and pathogenesis by regulating the PI3K/AKT signaling pathway. PTEN degradation activates the PI3K/AKT pathway and inhibits autophagy. These effects enhance macrophage migration and M2 polarization, thereby promoting tumor growth (78). NPC-Exo associated PD-1 and PTEN provide a basis for early biomarker screening and targeted therapy of nasopharyngeal carcinoma (**Figure 7**).

### 4.4 Melanoma cell-derived exosomes (MTEX)

MTEX contain the same antigens as the parental cells. Sharma et al. reported that exosomes isolated from the plasma of patients with melanoma contained melanoma-associated antigens compared with those isolated from normal cells. This evidence illustrates that plasma-derived exosomes from patients with melanoma may be a useful biomarker of melanoma in tumor liquid biopsy (259). Whiteside demonstrated that incubation of MTEX with immune-receptor cells resulted in inhibition of the anti-tumor function of these cells. MTEX are involved in immunosuppression in melanoma; therefore, they may play a role in promoting melanoma progression (342). Marton et al. purified and characterized B16F1 melanoma cell-derived exosomes. They found that MTEX affected the proliferation of CD4^+^T cells induced by bone marrow-derived DCs. MTEX also activated macrophages as measured by NF-κB activation. This finding suggests that exosomes play a role in tumor progression and metastasis formation by supporting tumor immune escape mechanisms (343). Gerloff et al. found that exposure to MTEX induced a tumor-promoting TAM phenotype. Sequencing showed that miR-125b-5p was enriched in MTEX. The miR-125b-5p could be delivered to macrophages through MTEX, targeted lysosomal acid lipase A (LIPA) in macrophages, and induced a tumor-promoting TAM phenotype (344).

### 4.5 Urologic tumor-derived exosomes

Xu et al. reported that exosomes derived from prostate cancer cells can transport IL-8 and bind to peroxisome proliferator activated receptor α (PPARα) in CD8^+^T cells (345). Through this process, they activate the uncoupling protein 1 (UCP1), decomcause fatty acids for thermogenesis, interfere with energy metabolism, hinder the function of CD8^+^T cells, and promote immune escape (345). Ding et al. studied exosomes derived from bladder cancer cell line T24; the exosomes blocked the function of NK cells by inhibiting the expression of NKG2D, NKP30, CD226, PRF, and GZMB receptors on NK cells, The miR-221–5p and miR-186–5p in exosomes derived from T24 cells interfere with the stable expression of DNAX-activation protein 10 (DAP10), CD96, and PRF mRNA in NK cells (346). Therefore, they may be targets for the treatment of bladder cancer (**Figure 7**).

### 4.6 Breast cancer cell-derived exosomes

Exosomal miR-20a-5p is released by breast cancer cells and transferred to CD8^+^ T cells, where it inhibits their function by targeting the nuclear protein coactivator of histone transcription (NPAT) (347). NPAT is a cell cycle gene highly expressed in immature CD8 T cells, The miR-20a-5p binds to the 3’-UTR of NPAT, inducing the development of resistance to anti-PD-1 therapy. These findings suggest that exosomal miR-20a-5p derived from triple-negative breast cancer plays an important role in promoting immune escape and immunotherapy resistance by inducing CD8^+^T cell dysfunction (347). Breast cancer-derived exosomes containing tumor cell-derived PD-L1 interact with PD-1-producing T cells to significantly reduce responses to immune checkpoint blockade agents. The drug macitane acts as a powerful helper of CD8^+^T cell anti-tumor response by inhibiting tumor cell-derived EV-PD-L1. Lee et al. reported that macitane inhibits the secretion of tumor-derived EV-PD-L1 in breast cancer cells by targeting endothelin receptor A (ETA) and can reduce the binding of PD-1 to EV-PD-L1, thereby synergizing the effect of anti-PD-L1 antibodies. Enhanced CD8^+^T cell-mediated tumor killing. These findings strongly support that macitane, which has been approved for clinical use, can be used to improve and/or overcome the inadequate response to PD-1/PD-L1 blockade therapy (348) (**Figure 7**).

### 4.7 Leukemia cell-derived exosomes (LEX)

CD4^+^T cells play a great role in tumor immunity, and can bind to DC-Exo generated by tumor cells to induce tumor immunity. Li et al. reported that LEX did not exert the expected effect when binding to CD4^+^T cells due to insufficient costimulatory ability, CD4^+^T-LEX-CD8086 was constructed, which could stimulate antigen-specific CD8^+^ cytotoxic T cell responses to leukemia cells. These data indicated that CD4^+^T cell vaccines using leukemia cell-derived exosomes modified by costimulatory molecules may be effective in immunotherapy for leukemia (200). Huang et al. reported that TGF-β1-silenced leukemia cell-derived exosomes (LEX-TGF-β1si) targeted DCs. The treatment effectively promoted the proliferation of CD4^+^T cells and the secretion of Th1 cytokines *in vitro*, and induced tumor-specific cytotoxic T cell responses to achieve an anti-leukemia effect. This evidence suggests that LEX-TGF-β1si targeting DCs are a promising immunotherapy option for leukemia (349) (**Figure 7**).

Due to the dual role of TEX in promoting tumor growth and as a therapeutic carrier, to overcome the tumor-promoting risk of TEX during treatment (324), immunotherapy combining immune system activation with immune escape inhibitors has been shown to be a new effective tumor suppression strategy, Fan et al. modified two antibodies (anti-PD-L1 and anti-CD40) on the surface of exosomes by co-culture. First, anti-PD-1 blocks immune checkpoint molecules by binding to the PD-L1 receptor on tumor cells. Second, anti-CD40 will direct exosomes to target the CD40 receptor on the membrane of dendritic cells (DC). After DC receives a positive costimulatory signal, exosomes will be taken up by DC and release cGAMP through lysosome-mediated exosome permeability to produce type I interferon (IFN-I) and pro-inflammatory cytokines. The two activation of dendritic cells (DCs) and the blocking of PD-L1 in tumor cells have improved the efficacy of combined cancer immunotherapy for tumor suppression (350). In addition to the use of dual agents, chimeric peptide exosomes are also a novel strategy for therapy, Cheng et al. engineered with chimeric peptides for dual plasma membrane and nuclear targeting photosensitizer delivery and synergistic photodynamic therapy (PDT), engineered chimeric peptide exosomes (ChiP-Exo) can achieve membrane targeting and, to some extent, lead to cell death, as the presence of nuclear localization signal (NLS) peptides can also enhance nuclear delivery. Nuclear ChiP-Exo activates reactive oxygen species (ROS) *in situ* to disrupt the nucleus, resulting in stable and synergistic PDT. This exosome-based dual-stage light-guided subcellular dual-target PDT strategy has shown greatly enhanced therapeutic effects in inhibiting tumor growth, providing new ideas for the development of individualized biomedicine for precise tumor treatment (351). Similarly, Trivedi et al. used dual-targeted hyaluronic acid nanoparticles to manipulate exosome contents by gene transfection into tumor cells. Studies have found that changes in miRNA levels in exosomes can mediate the repolarization of macrophages to a more pro-inflammatory/anti-tumor M1 phenotype, indicating that gene transfer of exosomes can support an anti-tumor environment, thereby reducing tumorigenesis (352).

Liquid biopsy is of great significance in the early diagnosis, treatment staging and prognosis of cancer (50). In recent years, tumor-derived exosomes (TEX) have become a popular biomarker and potential candidate for non-invasive liquid biopsy and diagnosis of a variety of cancers due to their tumor-specific antigen (TSA) (353). Blood is a commonly used specimen for testing, and future studies may focus on fluids other than blood (e.g., ascites, urine and cerebrospinal fluid, etc.) (354). Early biomarkers of ovarian cancer are limited, and it is difficult to detect ovarian cancer at an early stage due to the deep anatomical position of the ovary (355). Therefore, the study of exosomes provides a new method for the diagnosis of ovarian cancer (356). Cheng et al. reported the proteomic and lipidomic analysis of exosomes derived from ovarian cancer cells (SKOV-3) and ovarian surface epithelial cells (HOSEPiC) and found that Cholesterol Ester (ChE), Zymosterol (ZyE), V collagen alpha 2 chain (COL5A2) and lipoprotein lipase (LPL) than from HOSEPiC usually secrete body content is richer, therefore, outside the body protein and lipid secretion has certain application value in the early diagnosis of ovarian cancer (355). In addition, the popularization of liquid biopsy also provides a new idea for the diagnosis of thyroid cancer (PTC) (357). The diagnosis of PTC is generally performed by fine needle aspiration biopsy, but this method is limited in use, with low accuracy and tissue trauma (358). The circular RNA (circRNA) in exosomes has shown great value in cancer diagnosis (359). Dai et al. detected hsa_circ_0082002 and hsa_circ_0003863 in serum exosomes from healthy people, benign thyroid tumors and PTC without Hashimoto’s thyroiditis. It was found that the levels of exosomal hsa_circ_0082002 and hsa_circ_0003863 were positively correlated with lymph node metastasis and vascular invasion of PTC, Therefore, exosomal circRNA has the potential to be used as a tumor marker for the diagnosis of PTC (360). In summary, cancer cell-derived exosomes (glioma, nasopharyngeal carcinoma, liver, gastric, bladder, prostate, breast, and leukemia) provide new diagnostic methods and targets for the treatment of cancer, thereby potentially improving medical care.

## 5 Exosomes derived from other sources in cancer therapy

Munagala et al. reported that milk can be a source of exosomes (361). Milk fat globulus membrane (MFGM) proteins (i.e., butyrophilin, xanthan oxidase, adipophilin, and lactadherin) are the most abundant proteins found in milk-derived exosomes (362). These exosomes play various physiological and therapeutic roles in cell proliferation, inflammation, immune regulation, and cancer function, largely due to their cargo molecules (e.g., proteins, miRNAs) (363). Milk-derived exosomes are characterized by cross-species tolerance without adverse immune and inflammatory responses (361). Milk-derived exosomes demonstrate versatility in the cargo they carry, and have the ability to target tumors. Drug-loaded exosomes showed significantly higher efficacy against tumors *in vivo* compared with free drugs (361). Tumor-targeting ligands (e.g., folic acid) enhance the targeting of cancer cells by exosomes, resulting in better tumor killing. Milk derived exosomes are natural exosomes, their membranes contain hydrophilic and hydrophobic components, can load water-soluble and lipid-soluble drugs, have affinity with epithelial cells, and can be digested by cells through endocytosis mechanism. Therefore, it can be used for oral delivery (364). Cui et al. summarized the specific role of milk derived exosomes in the prevention and treatment of intestinal diseases. Milk derived exosomes can regulate intestinal immune homeostasis and restore the composition of intestinal flora, and play a role in intestinal diseases such as inflammatory bowel disease, necrotizing enterocolitis, and colorectal cancer (365). In addition, Yan et al. with milk source outside secrete body as miR-31–5p delivery tool, the study found that secrete outside body carrying miR-31–5p achieved higher cellular uptake, resistance to degradation, promote angiogenesis and diabetic wound healing in the body (366). However, to use it in the treatment of clinical diseases, higher concentration and purity are required, so a lot of *in vivo* clinical studies are still needed.

Exosome therapy, as an emerging therapeutic approach, plays a significant role in liquid biopsy (367, 368), cardiovascular and cerebrovascular diseases, wound healing (369), skin regeneration (370), neurodegenerative diseases, ocular diseases, skeletal diseases, and targeted therapy and diagnosis of tumors (371). Increasingly more research is gradually progressing towards clinical trials. Dang et al. reported that exosomes carrying LncRNA TUG1 were utilized for the treatment of myocardial infarction subsequently downregulating angiogenesis through the HIF-1α/VEGF-α axis. This effect could be counteracted by remote ischemic preconditioning (RIC), thereby demonstrating the potential therapeutic target of LncRNA TUG1 for myocardial infarction PCI reperfusion or non-reperfusion afterward (143). Additionally, Liang et al. discovered that LncRNA has a predictive role in coronary artery disease (CAD) with plasma exosomes encapsulated SOCS2-AS1 serving as an independent protective factor against CAD (372). Sun et al. developed a targeted treatment method for adhesive capsulitis by identifying differentially expressed miRNAs between patients with and without adhesive capsulitis, They found that miR-142 was significantly upregulated in the exosomes of adhesive capsulitis (Exo-S), both Exo-S and miR-142 inhibit fibrosis. The mechanism behind this action is that miR-142 can bind to transforming growth factor beta receptor 1 (Tgfbr1), By mimicking this biological function, liposomes loaded with si-Tgfbr1 can alleviate shoulder stiffness in a preclinical setting (373). Lenka et al. also investigated the lncRNA expression profile of serum exosomes in peripheral blood of healthy people, monoclonal gammopathy (MGUS) patients and multiple myeloma (MM) patients, and found that only one exosomal lncRNA PRINS was dysregulated in MM and healthy people. Show that secrete body outside the lncRNA PRINS in monoclonal ivig disease diagnosis (374).

At present, the research of exosomes mainly focuses on basic research, and only a small number of exosomes have been used in clinical trials. As the role of exosomes in the disease process becomes clearer, exosomes are increasingly being developed for disease treatment and diagnosis. Although there are no approved clinical exosome products, the number of ongoing clinical trials involving exosome-based therapies and diagnostics is increasing. When experimental research is transformed into clinical research, more attention should be paid to the convenience, stability and accuracy of isolation technology, as well as the requirements of exosome production, and to ensure high-quality large-scale production of exosomes. Although significant progress has been made in exosome isolation technology, none of the existing technologies is perfect, and sufficient clinical samples are needed to test the stability, safety, accuracy and convenience of each technology before being translated into clinical application. This process needs to be explored constantly. With the increasing improvement and maturity of technology, we believe that with the continuous efforts of researchers and scientific researchers, Better methods can be found for clinical application.

## 6 Conclusions

In this review, we introduce the functions and applications of exosomes obtained from different sources in cancer. The aim was to better understand the great potential of exosomes as drug carriers and diagnostic markers. Insight has been gained into the properties of exosomes derived from MSCs, DCs, macrophages, etc., providing more strategies for the treatment of tumors. The application of nanocarriers helps us overcome the limitations of traditional tumor treatment (e.g., chemotherapy resistance, inability to cross the BBB, damage to other healthy organs). The discovery of more effective methods for the treatment of tumors based on the available evidence is necessary to safeguard human health. However, the source, purification and characterization of evs are still limited for large-scale application. The sources and preparation methods of extracellular vesicles vary according to their sources. Before selecting a separation strategy, it is necessary to carefully consider the nature of the samples and research objectives in order to choose appropriate technical combinations. To ensure reproducibility and comparability of results, it is important to have consistent sample sources as a prerequisite. Furthermore, consistency in the methods used for extracellular vesicle isolation and characterization should be ensured. Different extraction methods such as centrifugation, precipitation, immunoprecipitation, and particle-based separation yield different results. For example, immunoprecipitation is preferred for plasma-derived samples due to their high viscosity which makes it difficult to obtain highly pure extracellular vesicles through other methods (375). Methods used for characterizing extracellular vesicles include transmission electron microscopy, nanoparticle tracking analysis, dynamic light scattering, flow cytometry, and immunohistochemical analysis which can be combined to characterize the morphology, biochemical composition, and receptors of extracellular vesicles. Only when there is consistency in the source selection method and acquisition conditions can a set of experiments be comparable. It is crucial to comprehensively study extracellular vesicle isolation protocols and standardize their characterization in order to ensure reproducibility and comparability. In order to guarantee the quality of secrete body source outside supervision, need more perfect preclinical studies, such as: different generation time of the stability of the outside source of MSC secrete body, tumor suppression, and need enough preclinical animal experiments and clinical I II, III period of study.

There are increasing preclinical studies on extracellular vesicles, including their role in neuro-related diseases (e.g., epilepsy, Parkinson’s disease, stroke), autoimmune diseases (e.g., rheumatoid arthritis, multiple sclerosis), skin regeneration, and wound healing. The emergence of extracellular vesicles in these areas undoubtedly provides a glimmer of hope for patients suffering from such conditions. However, there are still significant limitations to the direct clinical application of extracellular vesicles, such as individual variability, immune rejection reactions, and a lack of specific clinical efficacy studies. Therefore, the transition from preclinical research to clinical trials remains a challenging task that needs to be addressed. Nevertheless, with further advancements in extracellular vesicle research, these issues can be resolved and it is evident that extracellular vesicles hold great potential as a powerful therapeutic tool in various fields including tumor treatment, immune system disorders and neuro-related diseases. If we can solve the problems of production efficiency, limitation, and dosage of exosomes, the translation of exosomes from preclinical to clinical research is expected.

## Author contributions

XJ: Conceptualization, Writing – original draft, Writing – review & editing. JZ: Conceptualization, Data curation, Supervision, Writing – review & editing. YZ: Formal analysis, Project administration, Visualization, Writing – original draft. JH: Conceptualization, Investigation, Resources, Writing – original draft. MW: Conceptualization, Writing – original draft, Writing – review & editing. YH: Data curation, Formal analysis, Software, Writing – review & editing. SG: Data curation, Investigation, Methodology, Validation, Writing – original draft. XX: Data curation, Methodology, Software, Validation, Writing – review & editing. YL: Funding acquisition, Resources, Supervision, Writing – original draft.
